# Supplementary material for: Inferring causal pathways among three or more variables from steady-state correlations in a homeostatic system
Source: PLoS One. 2018 Oct 11;13(10):e0204755. doi: 10.1371/journal.pone.0204755 (PMC6181337; doi:10.1371/journal.pone.0204755)
Supplement: S1 Text — (DOCX) [file pone.0204755.s001.docx]

**Supporting Information**

**S1Text. Deriving predictions for regression parameters based on causal equations.**

**Acyclic pathways**

**Pathway P1: Linear**

**Prediction R1**

**Proof 1**

$$r_{\mathrm{AB}}=\frac{\sum e_{a}e_{b}}{\sqrt{\sum e_{a}^{2}}\sqrt{\sum e_{b}^{2}}}=\frac{\sum e_{a}(m_{1}e_{a}+e_{1})}{\sqrt{\sum e_{a}^{2}}\sqrt{\sum e_{b}^{2}}}=\frac{m_{1}\sum e_{a}^{2}}{\sqrt{\sum e_{a}^{2}}\sqrt{\sum e_{b}^{2}}}=\frac{m_{1}\sqrt{\sum e_{a}^{2}}}{\sqrt{\sum e_{b}^{2}}}$$

$$r_{\mathrm{BC}}=\frac{\sum e_{b}e_{c}}{\sqrt{\sum e_{b}^{2}}\sqrt{\sum e_{c}^{2}}}=\frac{\sum e_{b}(m_{2}e_{b}+e_{2})}{\sqrt{\sum e_{b}^{2}}\sqrt{\sum e_{c}^{2}}}=\frac{m_{2}\sum e_{b}^{2}}{\sqrt{\sum e_{b}^{2}}\sqrt{\sum e_{c}^{2}}}=\frac{m_{2}\sqrt{\sum e_{b}^{2}}}{\sqrt{\sum e_{c}^{2}}}$$

$$r_{\mathrm{AC}}\frac{\sum e_{a}e_{c}}{\sqrt{\sum e_{a}^{2}}\sqrt{\sum e_{c}^{2}}}=\frac{\sum e_{a}(m_{2}m_{1}e_{a}+m_{2}e_{1}+e_{2})}{\sqrt{\sum e_{a}^{2}}\sqrt{\sum e_{c}^{2}}}=\frac{m_{2}m_{1}\sum e_{a}^{2}}{\sqrt{\sum e_{a}^{2}}\sqrt{\sum e_{c}^{2}}}=\frac{m_{1}m_{2}\sqrt{\sum e_{a}^{2}}}{\sqrt{\sum e_{c}^{2}}}$$

Therefore

$r_{\mathrm{AC}}-r_{\mathrm{AB}}.r_{\mathrm{BC}}=m_{2}m_{1}\frac{\sigma e_{a}}{\sigma e_{c}}-m_{1}\frac{\sigma e_{a}}{\sigma e_{b}}.m_{2}\frac{\sigma e_{b}}{\sigma e_{c}}=0$.

**Alternative general proof**

There can be an alternative general proof for specific relationships between $r_{\mathrm{AB}}^{2}$, $r_{\mathrm{BC}}^{2}$ and $r_{\mathrm{AC}}^{2}$ that take different forms under different conditions and explain the predictions of all pathways.

Since $r^{2}$ is known to be equal to the variance explained and since,

$$\sigma^{2}B=M_{\mathrm{ba}}^{2}\sigma^{2}A+\sigma^{2}E_{\mathrm{ba}}$$

$$r_{\mathrm{AB}}^{2}.r_{\mathrm{BC}}^{2}=\frac{M_{\mathrm{ba}}^{2}\sigma^{2}A}{M_{\mathrm{ba}}^{2}\sigma^{2}A+\sigma^{2}E_{\mathrm{ba}}}.\frac{M_{\mathrm{cb}}^{2}\sigma^{2}B}{M_{\mathrm{CB}}^{2}\sigma^{2}B+\sigma^{2}E_{\mathrm{cb}}}$$

$r_{\mathrm{AB}}^{2}.r_{\mathrm{BC}}^{2}=\frac{M_{\mathrm{BA}}^{2}.M_{\mathrm{CB}}^{2}.\sigma^{2}A}{M_{\mathrm{BA}}^{2}.M_{\mathrm{CB}}^{2}.\sigma^{2}A+M_{\mathrm{CB}}^{2}\sigma^{2}E_{\mathrm{ba}}+\sigma^{2}E_{\mathrm{cb}}}$ …………….. [General Equation R1]

Specifically, for the linear pathway (see table P1), three condition namely (a) $M_{\mathrm{cb}}M_{\mathrm{ba}}= M_{\mathrm{ca}}$ (b) $M_{\mathrm{cb}}E_{\mathrm{ba}}+E_{\mathrm{cb}}=E_{\mathrm{ca}}$ and (c) there is no covariance between $E_{\mathrm{ba}}$and$E_{\mathrm{cb}}$ making

$$M_{\mathrm{CB}}^{2}\sigma^{2}E_{\mathrm{ba}}+\sigma^{2}E_{\mathrm{cb}}=\sigma^{2}E_{\mathrm{ca}}$$

Therefore $r_{\mathrm{AB}}^{2}.r_{\mathrm{BC}}^{2}=\frac{M_{\mathrm{ca}}^{2}.\sigma^{2}A}{M_{\mathrm{ca}}^{2}.\sigma^{2}A+\sigma^{2}E_{\mathrm{cA}}}=r_{\mathrm{AC}}^{2}$

**Pathway P2: Radiating**

The causal equations for this model would be

$$A= m_{1}B+e_{1}+k_{1}$$

$$B= input=\bar{B}+e_{b}$$

$$C= m_{2}B+e_{2}+k_{2}$$

Regression parameters can be derived from the causal equations as follows

$$e_{a}=m_{1}e_{b}+e_{1}$$

$$e_{b}=input variability$$

$$e_{c}=m_{2}e_{b}+e_{2}$$

$$M_{\mathrm{ba}}=\frac{\sum e_{a}e_{b}}{\sum e_{a}^{2}}=\frac{\sum(m_{1}e_{b}+e_{1})e_{b}}{\sum e_{a}^{2}}=\frac{m_{1}\sum e_{b}^{2}}{\sum e_{a}^{2}}=\frac{1}{m_{1}}\frac{m_{1}^{2}\sum e_{b}^{2}}{\sum e_{a}^{2}}=\frac{1}{m_{1}}r_{\mathrm{AB}}^{2}$$

$$M_{\mathrm{cb}}=\frac{\sum e_{c}e_{b}}{\sum e_{b}^{2}}=\frac{\sum(m_{2}e_{b}+e_{2})e_{b}}{\sum e_{b}^{2}}=\frac{m_{2}\sum e_{b}^{2}}{\sum e_{b}^{2}}=m_{2}$$

$$M_{\mathrm{ca}}=\frac{\sum e_{c}e_{a}}{\sum e_{a}^{2}}=\frac{\sum(m_{2}e_{b}+e_{2})(m_{1}e_{b}+e_{1})}{\sum e_{a}^{2}}=\frac{m_{2}m_{1}\sum e_{b}^{2}}{\sum e_{a}^{2}}=\frac{m_{2}}{m_{1}}\frac{m_{1}^{2}\sum e_{b}^{2}}{\sum e_{a}^{2}}=\frac{m_{2}}{m_{1}}r_{\mathrm{AB}}^{2}$$

$$E_{\mathrm{ba}}=e_{b}-M_{\mathrm{ba}}e_{a}=e_{b}-\frac{1}{m_{1}}r_{\mathrm{AB}}^{2}(m_{1}e_{b}+e_{1})=e_{b}(1-r_{\mathrm{AB}}^{2})-\frac{1}{m_{1}}r_{\mathrm{AB}}^{2}e_{1}$$

$$E_{\mathrm{cb}}=e_{c}-M_{\mathrm{cb}}e_{b}=m_{2}e_{b}+e_{2}-m_{2}e_{b}=e_{2}$$

$$E_{\mathrm{ca}}=e_{c}-M_{\mathrm{ca}}e_{a}=m_{2}e_{b}+e_{2}-\frac{m_{2}}{m_{1}}r_{\mathrm{AB}}^{2}(m_{1}e_{b}+e_{1})=m_{2}e_{b}(1-r_{\mathrm{AB}}^{2})-\frac{m_{2}}{m_{1}}r_{\mathrm{AB}}^{2}e_{1}+e_{2}$$

The relationships in table P2 in the main text are inferred from the above.

**Prediction R1**

$$r_{\mathrm{AB}}=\frac{\sum(e_{a})(e_{b})}{\sqrt{\sum e_{a}^{2}}\sqrt{\sum e_{b}^{2}}}=\frac{\sum(m_{1}e_{b}+e_{1})(e_{b})}{\sqrt{\sum e_{a}^{2}}\sqrt{\sum e_{b}^{2}}}=\frac{m_{1}\sqrt{\sum e_{b}^{2}}}{\sqrt{\sum e_{a}^{2}}}$$

$$r_{\mathrm{BC}}=\frac{\sum(e_{c})(e_{b})}{\sqrt{\sum e_{c}^{2}}\sqrt{\sum e_{b}^{2}}}=\frac{\sum(m_{2}e_{b}+e_{2})(e_{b})}{\sqrt{\sum e_{c}^{2}}\sqrt{\sum e_{b}^{2}}}=\frac{m_{2}\sqrt{\sum e_{b}^{2}}}{\sqrt{\sum e_{c}^{2}}}$$

$$r_{\mathrm{AC}}=\frac{\sum(e_{c})(e_{a})}{\sqrt{\sum e_{c}^{2}}\sqrt{\sum e_{a}^{2}}}=\frac{\sum(m_{2}e_{b}+e_{2})(m_{1}e_{b}+e_{1})}{\sqrt{\sum e_{c}^{2}}\sqrt{\sum e_{a}^{2}}}=\frac{m_{2}m_{1}\sum e_{b}^{2}}{\sqrt{\sum e_{c}^{2}}\sqrt{\sum e_{a}^{2}}}$$

Therefore

$$r_{\mathrm{AC}}-r_{\mathrm{AB}}.r_{\mathrm{BC}}=\frac{m_{2}m_{1}\sum e_{b}^{2}}{\sqrt{\sum e_{c}^{2}}\sqrt{\sum e_{a}^{2}}}-\frac{m_{1}\sqrt{\sum e_{b}^{2}}}{\sqrt{\sum e_{a}^{2}}}.\frac{m_{2}\sqrt{\sum e_{b}^{2}}}{\sqrt{\sum e_{c}^{2}}}=0$$

Alternatively, from the general equation R1 the three conditions namely (a) $M_{\mathrm{cb}}M_{\mathrm{ba}}= M_{\mathrm{ca}}$ (b) $M_{\mathrm{cb}}E_{\mathrm{ba}}+E_{\mathrm{cb}}=E_{\mathrm{ca}}$ and (c) there is no covariance between $E_{\mathrm{ba}}$ and $E_{\mathrm{cb}}$ are satisfied.

Therefore $r_{\mathrm{AB}}^{2}.r_{\mathrm{BC}}^{2}=\frac{M_{\mathrm{ca}}^{2}.\sigma^{2}A}{M_{\mathrm{ca}}^{2}.\sigma^{2}A+\sigma^{2}E_{\mathrm{cA}}}=r_{\mathrm{AC}}^{2}$

**Prediction R2**

It is clear from table P2 that

$$M_{\mathrm{ca}}-M_{\mathrm{cb}}M_{\mathrm{ba}}=\frac{m_{2}}{m_{1}}r_{\mathrm{AB}}^{2}-m_{2}.\frac{1}{m_{1}}r_{\mathrm{AB}}^{2}=0$$

Note that the regression slopes $M_{\mathrm{ca}}$ and $M_{\mathrm{ba}}$ will be underestimates as compared to the true slope of the relationship since there is likely to be post effect error in A. However, since both the slopes will be underestimated by the same factor that is $r_{\mathrm{AB}}^{2}$, $M_{\mathrm{ca}}$ can be predicted accurately by the product of the two slopes $M_{\mathrm{cb}}$ and $M_{\mathrm{ba}}$.

**Prediction R3**

From the table since $e_{1}$ and $e_{2}$ are not correlated, no correlation is expected between $E_{\mathrm{ba}}$ and $E_{\mathrm{cb}}$. Underestimation of the slope $M_{\mathrm{ba}}$ can introduce a significant correlation between $E_{\mathrm{ba}}$ and B but since $E_{\mathrm{cb}}$ is uncorrelated with B, the prediction of no correlation between $E_{\mathrm{ba}}$ and $E_{\mathrm{cb}}$ is not affected.

$$r_{E_{\mathrm{ba}}E_{\mathrm{cb}}}=r_{(e_{b}(1-r_{\mathrm{AB}}^{2})-\frac{1}{m_{1}}r_{\mathrm{AB}}^{2}e_{1})(e_{2})}=0$$

**Prediction R4**

Intuitively since $e_{1}$ is independent of B, no correlation is expected between $E_{\mathrm{ba}}$ and C. This means that correcting B for A should completely destroy its correlation with C. However here we assume that the correction is made with the true slopes. Since B is causal to A, there will be post-effect error in A which would underestimate the slope of regression of B on A. Because of the underestimation bias, estimated $E_{\mathrm{ba}}$ will have a correlation with B and since B is correlated to C there can be a residual correlation between $E_{\mathrm{ba}}$ and C. Since the underestimation is a function of the post effect error which decides $r_{\mathrm{AB}}^{2}$, $r_{\mathrm{AB}}^{2}$ could be a good predictor of the proportionate decrease in squared correlation coefficient. Simulations show (see below) that the relationship between the proportionate decrease with $r_{\mathrm{AB}}^{2}$ cannot be differentiated from that of P1.

**Proof 2**

$$r_{\mathrm{BC}}=\frac{m_{2}\sqrt{\sum e_{b}^{2}}}{\sqrt{\sum e_{c}^{2}}}$$

$$r_{E_{\mathrm{ba}}C}=\frac{m_{2}(1-r_{\mathrm{AB}}^{2})\sum e_{b}^{2}}{\sqrt{\sum E_{\mathrm{ba}}^{2}}\sqrt{\sum e_{c}^{2}}}$$

$$r_{E_{\mathrm{ba}}C}=(1-r_{\mathrm{AB}}^{2})\frac{\sqrt{\sum e_{b}^{2}}}{\sqrt{\sum E_{\mathrm{ba}}^{2}}}r_{\mathrm{BC}}$$

As ${0<r}_{\mathrm{AB}}^{2}<1$ and $\sum E_{\mathrm{ba}}^{2}<\sum e_{b}^{2}$ since the total variability in B is greater than the residuals of the regression of B on A,

$$r_{E_{\mathrm{ba}}C}<r_{\mathrm{BC}}$$

It follows that the proportionate difference in $r_{E_{\mathrm{ba}}C}^{2}$ and $r_{E_{\mathrm{ba}}C}^{2}$, i.e.

$$\frac{r_{\mathrm{BC}}^{2}-r_{E_{\mathrm{ba}}C}^{2}}{r_{\mathrm{BC}}^{2}}=1-\frac{r_{E_{\mathrm{ba}}C}^{2}}{r_{\mathrm{BC}}^{2}}$$

$$=1-\left( \frac{m_{2}(1-r_{\mathrm{AB}}^{2})\sum e_{b}^{2}}{\sqrt{\sum e_{E_{\mathrm{ba}}}^{2}}\sqrt{\sum e_{c}^{2}}}/\frac{m_{2}\sqrt{\sum e_{b}^{2}}}{\sqrt{\sum e_{c}^{2}}} \right)^{2}$$

$$=1-\left( \frac{(1-r_{\mathrm{AB}}^{2})\sqrt{\sum e_{b}^{2}}}{\sqrt{\sum E_{\mathrm{ba}}^{2}}} \right)^{2}=1-\left( \frac{\sqrt{\sum e_{1}^{2}}}{\sqrt{\sum e_{a}^{2}}} \right)^{2}=r_{\mathrm{AB}}^{2}$$

**Pathway P3: convergent:**

The causal equations for this model would be

$$A=input=\bar{A}+e_{a}$$

$$B=m_{1}A+m_{2}C+e_{1}+k_{1}$$

$$C=input=\bar{C}+e_{c}$$

Where $e_{a}$, $e_{1}$ and $e_{c}$ are uncorrelated

Regression parameters can be derived from the causal equations as follows

$$e_{a}=input variability$$

$$e_{b}=m_{1}e_{a}+m_{2}e_{c}+e_{1}$$

$$e_{c}=input variability$$

$$M_{\mathrm{ba}}=\frac{\sum e_{a}e_{b}}{\sum e_{a}^{2}}=\frac{\sum e_{a}(m_{1}e_{a}+m_{2}e_{c}+e_{1})}{\sum e_{a}^{2}}=\frac{m_{1}\sum e_{a}^{2}}{\sum e_{a}^{2}}=m_{1}$$

$$M_{\mathrm{cb}}=\frac{\sum e_{c}e_{b}}{\sum e_{b}^{2}}=\frac{\sum e_{c}(m_{1}e_{a}+m_{2}e_{c}+e_{1})}{\sum e_{b}^{2}}=\frac{m_{2}\sum e_{c}^{2}}{\sum e_{b}^{2}}=\frac{1}{m_{2}}\frac{m_{2}^{2}\sum e_{c}^{2}}{\sum e_{b}^{2}}=\frac{1}{m_{2}}r_{\mathrm{BC}}^{2}$$

$M_{\mathrm{ca}}=\frac{\sum e_{c}e_{a}}{\sum e_{a}^{2}}=0$ since there is no covariance between e_c_ and e_a_

$$E_{\mathrm{ba}}=e_{b}-M_{\mathrm{ba}}e_{a}=m_{1}e_{a}+m_{2}e_{c}+e_{1}-m_{1}e_{a}=e_{1}+m_{2}e_{c}$$

$$E_{\mathrm{cb}}=e_{c}-M_{\mathrm{cb}}e_{b}=e_{c}-\frac{1}{m_{2}}r_{\mathrm{BC}}^{2}(m_{1}e_{a}+m_{2}e_{c}+e_{1})=e_{c}(1-r_{\mathrm{BC}}^{2})-\frac{1}{m_{2}}r_{\mathrm{BC}}^{2}(m_{1}e_{a}+e_{1})$$

$$E_{\mathrm{ca}}=e_{c}-M_{\mathrm{ca}}e_{a}=e_{c}-0=e_{c}$$

The relationships in table P3 in the main text are inferred from the above.

**Prediction R1**

$$r_{\mathrm{AB}}=\frac{\sum e_{a}e_{b}}{\sqrt{\sum e_{a}^{2}}\sqrt{\sum e_{b}^{2}}}=\frac{\sum e_{a}(m_{1}e_{a}+m_{2}e_{c}+e_{1})}{\sqrt{\sum e_{a}^{2}}\sqrt{\sum e_{b}^{2}}}=\frac{m_{1}\sum e_{a}^{2}}{\sqrt{\sum e_{a}^{2}}\sqrt{\sum e_{b}^{2}}}=m_{1}\frac{\sqrt{\sum e_{a}^{2}}}{\sqrt{\sum e_{b}^{2}}}$$

$$r_{\mathrm{BC}}=\frac{\sum(e_{b})(e_{c})}{\sqrt{\sum e_{b}^{2}}\sqrt{\sum e_{c}^{2}}}=\frac{\sum(m_{1}e_{a}+m_{2}e_{c}+e_{1})(e_{c})}{\sqrt{\sum e_{b}^{2}}\sqrt{\sum e_{c}^{2}}}=\frac{m_{2}\sum e_{c}^{2}}{\sqrt{\sum e_{b}^{2}}\sqrt{\sum e_{c}^{2}}}=\frac{m_{2}\sqrt{\sum e_{c}^{2}}}{\sqrt{\sum e_{b}^{2}}}$$

$r_{\mathrm{AC}}=\frac{\sum(e_{a})(e_{c})}{\sqrt{\sum e_{a}^{2}}\sqrt{\sum e_{c}^{2}}}=0$

As long as $r_{\mathrm{AB}}$and $r_{\mathrm{BC}}$ are non zero, $r_{\mathrm{AB}}^{2}.r_{\mathrm{BC}}^{2}>r_{\mathrm{AC}}^{2}$

Alternatively from the general equation 1,

$$r_{\mathrm{AB}}^{2}.r_{\mathrm{BC}}^{2}=\frac{M_{\mathrm{ba}}^{2}.M_{\mathrm{cb}}^{2}.\sigma^{2}A}{M_{\mathrm{ba}}^{2}.M_{\mathrm{cb}}^{2}.\sigma^{2}A+M_{\mathrm{cb}}^{2}\sigma^{2}E_{\mathrm{ba}}+\sigma^{2}E_{\mathrm{cb}}}$$

Since $M_{\mathrm{ca}}^{2}<M_{\mathrm{ba}}^{2}.M_{\mathrm{cb}}^{2}$, and there is positive covariance between E_ba_ and E_cb_

$\frac{M_{\mathrm{ba}}^{2}.M_{\mathrm{cb}}^{2}.\sigma^{2}A}{M_{\mathrm{ba}}^{2}.M_{\mathrm{cb}}^{2}.\sigma^{2}A+M_{\mathrm{cb}}^{2}\sigma^{2}E_{\mathrm{ba}}+\sigma^{2}E_{\mathrm{cb}}}$ >

$$r_{\mathrm{AC}}^{2}-r_{\mathrm{AB}}^{2}.r_{\mathrm{BC}}^{2}<0$$

**Prediction R2**

Since the expected slope $M_{\mathrm{ca}}$ is zero and both $M_{\mathrm{ba}}$ and $M_{\mathrm{cb}}$ non-zero, their product is not a predictor of $M_{\mathrm{ca}}$.

$\left| M_{\mathrm{ca}} \right|-\left| M_{\mathrm{cb}}M_{\mathrm{ba}} \right|<0$ as $\left| M_{\mathrm{ca}} \right|=0$

**Prediction R3**

$$r_{E_{\mathrm{ba}}E_{\mathrm{cb}}}=\frac{\sum E_{\mathrm{ba}}E_{\mathrm{cb}}}{\sqrt{\sum E_{\mathrm{ba}}^{2}}\sqrt{\sum E_{\mathrm{cb}}^{2}}}=\frac{\sum\left( e_{1}+m_{2}e_{c} \right)\left( e_{c}(1-r_{\mathrm{BC}}^{2})-\frac{1}{m_{2}}r_{\mathrm{BC}}^{2}(m_{1}e_{a}+e_{1}) \right)}{\sqrt{\sum E_{\mathrm{ba}}^{2}}\sqrt{\sum E_{\mathrm{cb}}^{2}}}$$

$$=\frac{m_{2}(1-r_{\mathrm{BC}}^{2})\sum e_{c}^{2}-\frac{1}{m_{2}}r_{\mathrm{BC}}^{2}\sum e_{1}^{2}}{\sqrt{\sum E_{\mathrm{ba}}^{2}}\sqrt{\sum E_{\mathrm{cb}}^{2}}}=\frac{m_{2}\left( 1-\frac{m_{2}^{2}\sum e_{c}^{2}}{\sum e_{b}^{2}} \right)\sum e_{c}^{2}-\frac{1}{m_{2}}\frac{m_{2}^{2}\sum e_{c}^{2}}{\sum e_{b}^{2}}\sum e_{1}^{2}}{\sqrt{\sum E_{\mathrm{ba}}^{2}}\sqrt{\sum E_{\mathrm{cb}}^{2}}}$$

$$=\frac{m_{2}\left( \frac{m_{1}^{2}\sum e_{a}^{2}+m_{2}^{2}\sum e_{c}^{2}+\sum e_{1}^{2}-m_{2}^{2}\sum e_{c}^{2}}{\sum e_{b}^{2}} \right)\sum e_{c}^{2}-\frac{m_{2}\sum e_{c}^{2}}{\sum e_{b}^{2}}\sum e_{1}^{2}}{\sqrt{\sum E_{\mathrm{ba}}^{2}}\sqrt{\sum E_{\mathrm{cb}}^{2}}}$$

$$=\frac{m_{2}\left( \frac{m_{1}^{2}\sum e_{a}^{2}+\sum e_{1}^{2}}{\sum e_{b}^{2}} \right)\sum e_{c}^{2}-\frac{m_{2}\sum e_{c}^{2}}{\sum e_{b}^{2}}\sum e_{1}^{2}}{\sqrt{\sum E_{\mathrm{ba}}^{2}}\sqrt{\sum E_{\mathrm{cb}}^{2}}}$$

$$=\frac{m_{2}\left( \frac{m_{1}^{2}\sum e_{a}^{2}}{\sum e_{b}^{2}} \right)\sum e_{c}^{2}}{\sqrt{\sum E_{\mathrm{ba}}^{2}}\sqrt{\sum E_{\mathrm{cb}}^{2}}}$$

Thus the sign of the correlation will be decided by the sign of the slope m_2_. In reality we will not have access to $m_{2}$ in data. However since $m_{2}$ is the sole factor deciding the sign of $M_{\mathrm{cb}}$, the prediction is that the sign of $r_{E_{\mathrm{ba}}E_{\mathrm{cb}}}$and the sign of $M_{\mathrm{cb}}$ should be the same. For similar reasons we can expect $r_{E_{\mathrm{bc}}E_{\mathrm{ab}}}$ to have the same sign as that of $M_{\mathrm{ba}}$.

**Prediction R4**

a.) Since both A and C influence B independently in this pathway, A contributes to the error in $r_{\mathrm{BC}}$ and C contributes to the error in $r_{\mathrm{AB}}$. Therefore, correcting for A improves its correlation with C and vice versa.

Formally

$r_{\mathrm{BC}}=\frac{m_{2}\sqrt{\sum e_{c}^{2}}}{\sqrt{\sum e_{b}^{2}}}$

$r_{E_{\mathrm{ba}}C}=\frac{m_{2}\sqrt{\sum e_{c}^{2}}}{\sqrt{\sum E_{\mathrm{ba}}^{2}}}$

Since $\sqrt{\sum e_{b}^{2}}>\sqrt{\sum E_{\mathrm{ba}}^{2}}$

$r_{\mathrm{BC}}<r_{E_{\mathrm{ba}}C}$

b.) Further it can be shown that the proportionate improvement in correlation after correcting for A is predicted well by $r_{\mathrm{AB}}^{2}$

$$\frac{r_{E_{\mathrm{ba}}C}^{2}-r_{\mathrm{BC}}^{2}}{r_{E_{\mathrm{ba}}C}^{2}}=1-\frac{r_{\mathrm{BC}}^{2}}{r_{E_{\mathrm{ba}}C}^{2}}=1-\frac{\sum E_{\mathrm{ba}}^{2}}{\sum e_{b}^{2}}=1-\frac{\sum E_{\mathrm{ba}}^{2}}{m_{1}^{2}\sum e_{a}^{2}+\sum E_{\mathrm{ba}}^{2}}=\frac{m_{1}^{2}\sum e_{a}^{2}}{\sum e_{b}^{2}}=r_{\mathrm{AB}}^{2}$$

Because this expression differs from R4a of the earlier pathways, we can use a more generalized and robust form for R4b as $\frac{\left| r_{\mathrm{BC}}^{2} -r_{E_{\mathrm{ba}}C}^{2} \right|}{\max\left( r_{\mathrm{BC}}^{2}, r_{E_{\mathrm{ba}}C}^{2} \right)}=r_{\mathrm{AB}}^{2}$

**Pathway P4: Common cause**

The causal equations for this model would be

$$X=input=\bar{X}+e_{x}$$

$$A=m_{1}X+e_{1}+k_{1}$$

$$B=m_{2}X+e_{2}+k_{2}$$

$C=m_{3}X+e_{3}+k_{3}$

where $e_{x}e_{1} e_{2}e_{3}$ are not correlated.

The regression parameters can be derived from the causal equations as follows.

$$e_{x}=input variability$$

$$e_{a}=m_{1}e_{x}+e_{1}$$

$$e_{b}=m_{2}e_{x}+e_{2}$$

$$e_{c}=m_{3}e_{x}+e_{3}$$

$$M_{\mathrm{ba}}=\frac{\sum(e_{a})(e_{b})}{\sum e_{a}^{2}}=\frac{\sum(m_{1}e_{x}+e_{1})(m_{2}e_{x}+e_{2})}{\sum e_{a}^{2}}=\frac{m_{1}m_{2}\sum e_{x}^{2}}{\sum e_{a}^{2}}=\frac{m_{2}}{m_{1}}\frac{m_{1}^{2}\sum e_{x}^{2}}{\sum e_{a}^{2}}=\frac{m_{2}}{m_{1}}r_{\mathrm{AX}}^{2}$$

$$M_{\mathrm{cb}}=\frac{m_{3}}{m_{2}}r_{\mathrm{BX}}^{2}$$

$$M_{\mathrm{ca}}=\frac{m_{3}}{m_{1}}r_{\mathrm{AX}}^{2}$$

All the three slopes underestimate the true slopes by $r_{\mathrm{AX}}^{2}, r_{\mathrm{BX}}^{2}\mathrm{and}r_{\mathrm{AX}}^{2}$ respectively.

$$E_{\mathrm{ba}}=e_{b}-M_{\mathrm{ba}}e_{a}=m_{2}e_{x}+e_{2}-\frac{m_{2}}{m_{1}}r_{\mathrm{AX}}^{2}(m_{1}e_{x}+e_{1})=m_{2}e_{x}(1-r_{\mathrm{AX}}^{2})+e_{2}-r_{\mathrm{AX}}^{2}\frac{m_{2}}{m_{1}}e_{1}$$

$$E_{\mathrm{cb}}=e_{c}-M_{\mathrm{cb}}e_{b}=m_{3}e_{x}(1-r_{\mathrm{BX}}^{2})+e_{3}-r_{\mathrm{BX}}^{2}\frac{m_{3}}{m_{2}}e_{2}$$

$$E_{\mathrm{ca}}=e_{c}-M_{\mathrm{ca}}e_{a}=m_{3}e_{x}(1-r_{\mathrm{AX}}^{2})+e_{3}-r_{\mathrm{AX}}^{2}\frac{m_{3}}{m_{1}}e_{1}$$

The relationships in table P4 in the main text are inferred from the above.

**Prediction R1**

$$r_{\mathrm{AX}}=\frac{\sum(e_{a})(e_{x})}{\sqrt{\sum e_{a}^{2}}\sqrt{\sum e_{x}^{2}}}=\frac{\sum(m_{1}e_{x}+e_{1})e_{x}}{\sqrt{\sum e_{a}^{2}}\sqrt{\sum e_{x}^{2}}}=\frac{m_{1}\sum e_{x}^{2}}{\sqrt{\sum e_{a}^{2}}\sqrt{\sum e_{x}^{2}}}=\frac{m_{1}\sqrt{\sum e_{x}^{2}}}{\sqrt{\sum e_{a}^{2}}}$$

$$r_{\mathrm{AB}}=\frac{\sum(e_{a})(e_{b})}{\sqrt{\sum e_{a}^{2}}\sqrt{\sum e_{b}^{2}}}=\frac{\sum(m_{1}e_{x}+e_{1})(m_{2}e_{x}+e_{2})}{\sqrt{\sum e_{a}^{2}}\sqrt{\sum e_{b}^{2}}}=\frac{m_{1}m_{2}\sum e_{x}^{2}}{\sqrt{\sum e_{a}^{2}}\sqrt{\sum e_{b}^{2}}}$$

$$r_{\mathrm{BC}}=\frac{\sum(e_{c})(e_{b})}{\sqrt{\sum e_{c}^{2}}\sqrt{\sum e_{b}^{2}}}=\frac{\sum(m_{3}e_{x}+e_{3})(m_{2}e_{x}+e_{2})}{\sqrt{\sum e_{c}^{2}}\sqrt{\sum e_{b}^{2}}}=\frac{m_{3}m_{2}\sum e_{x}^{2}}{\sqrt{\sum e_{c}^{2}}\sqrt{\sum e_{b}^{2}}}$$

$$r_{\mathrm{AC}}=\frac{\sum(e_{c})(e_{a})}{\sqrt{\sum e_{c}^{2}}\sqrt{\sum e_{a}^{2}}}=\frac{\sum(m_{3}e_{x}+e_{3})(m_{1}e_{x}+e_{1})}{\sqrt{\sum e_{c}^{2}}\sqrt{\sum e_{a}^{2}}}=\frac{m_{3}m_{1}\sum e_{x}^{2}}{\sqrt{\sum e_{c}^{2}}\sqrt{\sum e_{a}^{2}}}$$

$$r_{\mathrm{AC}}^{2}-r_{\mathrm{AB}}^{2}.r_{\mathrm{BC}}^{2}=\left( \frac{m_{3}m_{1}\sum e_{x}^{2}}{\sqrt{\sum e_{c}^{2}}\sqrt{\sum e_{a}^{2}}} \right)^{2}-\left( \frac{m_{1}m_{2}\sum e_{x}^{2}}{\sqrt{\sum e_{a}^{2}}\sqrt{\sum e_{b}^{2}}}.\frac{m_{3}m_{2}\sum e_{x}^{2}}{\sqrt{\sum e_{c}^{2}}\sqrt{\sum e_{b}^{2}}} \right)^{2}$$

$$=\left( \frac{m_{3}m_{1}\sum e_{x}^{2}}{\sqrt{\sum e_{c}^{2}}\sqrt{\sum e_{a}^{2}}} \right)^{2}\left( 1-\left( \frac{m_{2}^{2}\sum e_{x}^{2}}{\sum e_{b}^{2}} \right)^{2} \right)>0$$

as $e_{b}>m_{2}e_{x}$

Alternatively in the general equation R1 since there is negative covariance between $E_{\mathrm{ba}}$ and $E_{\mathrm{cb}}$,

$$M_{\mathrm{cb}}^{2}\sigma^{2}E_{\mathrm{ba}}+\sigma^{2}E_{\mathrm{cb}}>\sigma^{2}E_{\mathrm{ca}}$$

Therefore

$\frac{M_{\mathrm{ba}}^{2}.M_{\mathrm{cb}}^{2}.\sigma^{2}A}{M_{\mathrm{ba}}^{2}.M_{\mathrm{cb}}^{2}.\sigma^{2}A+M_{\mathrm{cb}}^{2}\sigma^{2}E_{\mathrm{ba}}+\sigma^{2}E_{\mathrm{cb}}}$ < $\frac{M_{\mathrm{ca}}^{2}.\sigma^{2}A}{M_{\mathrm{ca}}^{2}.\sigma^{2}A+\sigma^{2}E_{\mathrm{cA}}}$

$$r_{\mathrm{AC}}^{2}>r_{\mathrm{AB}}^{2}.r_{\mathrm{BC}}^{2}$$

${\mathrm{or} r}_{\mathrm{AC}}^{2}-r_{\mathrm{AB}}^{2}.r_{\mathrm{BC}}^{2}>0$

**Prediction R2**

In this pathway all the three variables A, B and C are likely to have post-effect errors and therefore each of the regression slopes will have a different underestimate bias. The error in A will contribute to the underestimation of both $M_{\mathrm{ca}}$ and $M_{\mathrm{ba}}$ by the same factor. But since $M_{\mathrm{cb}}$ will also be an underestimate, we expect . This inequality will be proportional to the post effect error in B. Error in B is extremely crucial for the definition of this pathway. If error in B is very small B is tightly correlated with X because of which this pathway tends to behave like the radiating pathway and the predictions of the radiating pathway work so that $\left| M_{\mathrm{ca}} \right|=\left| M_{\mathrm{cb}}M_{\mathrm{ba}} \right|$.

Formally

$$|M_{\mathrm{ca}}|-|M_{\mathrm{cb}}M_{\mathrm{ba}}|=|\frac{m_{3}}{m_{1}}r_{\mathrm{AX}}^{2}|-|\frac{m_{3}}{m_{2}}r_{\mathrm{BX}}^{2}.\frac{m_{2}}{m_{1}}r_{\mathrm{AX}}^{2}|=|\frac{m_{3}}{m_{1}}r_{\mathrm{AX}}^{2}|\left( |1|-|r_{\mathrm{BX}}^{2}| \right)>0$$

In the extreme case when error in B is small $r_{\mathrm{BX}}^{2}\to1$ and therefore $|M_{\mathrm{ca}}|-|M_{\mathrm{cb}}M_{\mathrm{ba}}|=0$

**Prediction R3**

$$r_{E_{\mathrm{ba}}E_{\mathrm{cb}}}=\frac{\sum E_{\mathrm{ba}}E_{\mathrm{cb}}}{\sqrt{\sum E_{\mathrm{ba}}^{2}}\sqrt{\sum E_{\mathrm{cb}}^{2}}}=\frac{\sum\left( m_{2}e_{x}(1-r_{\mathrm{AX}}^{2})+e_{2}-r_{\mathrm{AX}}^{2}\frac{m_{2}}{m_{1}}e_{1} \right)\left( m_{3}e_{x}(1-r_{\mathrm{BX}}^{2})+e_{3}-r_{\mathrm{BX}}^{2}\frac{m_{3}}{m_{2}}e_{2} \right)}{\sqrt{\sum E_{\mathrm{ba}}^{2}}\sqrt{\sum E_{\mathrm{cb}}^{2}}}$$

$$=\frac{m_{2}m_{3}(1-r_{\mathrm{AX}}^{2})(1-r_{\mathrm{BX}}^{2})\sum e_{x}^{2}-r_{\mathrm{BX}}^{2}\frac{m_{3}}{m_{2}}\sum e_{2}^{2}}{\sqrt{\sum E_{\mathrm{ba}}^{2}}\sqrt{\sum E_{\mathrm{cb}}^{2}}}=\frac{m_{2}m_{3}\left( 1-\frac{m_{1}^{2}\sum e_{x}^{2}}{\sum e_{a}^{2}} \right)\left( 1-\frac{m_{2}^{2}\sum e_{x}^{2}}{\sum e_{b}^{2}} \right)\sum e_{x}^{2}-\frac{m_{2}^{2}\sum e_{x}^{2}}{\sum e_{b}^{2}}\frac{m_{3}}{m_{2}}\sum e_{2}^{2}}{\sqrt{\sum E_{\mathrm{ba}}^{2}}\sqrt{\sum E_{\mathrm{cb}}^{2}}}$$

$$=\frac{m_{2}m_{3}\left( \frac{\sum e_{1}^{2}}{\sum e_{a}^{2}} \right)\left( \frac{\sum e_{2}^{2}}{\sum e_{b}^{2}} \right)\sum e_{x}^{2}-m_{2}m_{3}\frac{\sum e_{x}^{2}}{\sum e_{b}^{2}}\sum e_{2}^{2}}{\sqrt{\sum E_{\mathrm{ba}}^{2}}\sqrt{\sum E_{\mathrm{cb}}^{2}}}=\frac{m_{2}m_{3}\sum e_{x}^{2}\frac{\sum e_{2}^{2}}{\sum e_{b}^{2}}\left( \frac{\sum e_{1}^{2}}{\sum e_{a}^{2}}-1 \right)}{\sqrt{\sum E_{\mathrm{ba}}^{2}}\sqrt{\sum E_{\mathrm{cb}}^{2}}}$$

Since $\sum e_{a}^{2}>\sum e_{1}^{2}$, the bracket has a negative sign. Therefore the sign of the correlation is decided by the signs of $m_{1}$ and $m_{2}$. When both have the same signs $r_{E_{\mathrm{ba}}E_{\mathrm{cb}}}=-ve$ and when they have opposing signs $r_{E_{\mathrm{ba}}E_{\mathrm{cb}}}=+ve$. Because of this the correlation multiplied by the sign of $M_{\mathrm{ca}}$ is always negative.

**Prediction R4**

1. Since A is correlated to the common cause X, correcting for A can undermine the correlation between B and C.

Formally

$$r_{E_{\mathrm{ba}}C}=\frac{\sum(E_{\mathrm{ba}})(e_{c})}{\sqrt{\sum E_{\mathrm{ba}}^{2}}\sqrt{\sum e_{c}^{2}}}=\frac{\sum(m_{2}e_{x}(1-r_{\mathrm{AX}}^{2})+e_{2}-r_{\mathrm{AX}}^{2}\frac{m_{2}}{m_{1}}e_{1})(m_{3}e_{x}+e_{3})}{\sqrt{\sum E_{\mathrm{ba}}^{2}}\sqrt{\sum e_{c}^{2}}}=\frac{m_{2}m_{3}(1-r_{\mathrm{AX}}^{2})\sum e_{x}^{2}}{\sqrt{\sum E_{\mathrm{ba}}^{2}}\sqrt{\sum e_{c}^{2}}}$$

$$r_{\mathrm{BC}}=\frac{\sum(e_{c})(e_{b})}{\sqrt{\sum e_{c}^{2}}\sqrt{\sum e_{b}^{2}}}=\frac{m_{3}m_{2}\sum e_{x}^{2}}{\sqrt{\sum e_{c}^{2}}\sqrt{\sum e_{b}^{2}}}$$

So $r_{E_{\mathrm{ba}}C}=\left( 1-r_{\mathrm{AX}}^{2} \right)\frac{\sqrt{\sum e_{b}^{2}}}{\sqrt{\sum E_{\mathrm{ba}}^{2}}}r_{\mathrm{BC}}=\frac{(1-r_{\mathrm{AX}}^{2})}{(1-r_{\mathrm{AB}}^{2})}r_{\mathrm{BC}}$

Since $r_{\mathrm{AB}}^{2}$ < $r_{\mathrm{AX}}^{2}$, the denominator is greater than the numerator, therefore $r_{E_{\mathrm{ba}}C}<r_{\mathrm{BC}}$

1. Since, $r_{E_{\mathrm{ba}}C}<r_{\mathrm{BC}}$ we take

$$\frac{r_{\mathrm{BC}}^{2}-r_{E_{\mathrm{ba}}C}^{2}}{r_{\mathrm{BC}}^{2}}=1-\frac{r_{E_{\mathrm{ba}}C}^{2}}{r_{\mathrm{BC}}^{2}}=1-\frac{\left( 1-r_{\mathrm{AX}}^{2} \right)^{2}}{\left( 1-r_{\mathrm{AB}}^{2} \right)}$$

Now we know that $r_{\mathrm{AB}}^{2}<r_{\mathrm{AX}}^{2}$therefore

$$\left( 1-r_{\mathrm{AB}}^{2} \right)^{2}>\left( 1-r_{\mathrm{AX}}^{2} \right)^{2}$$

$$\left( 1-r_{\mathrm{AB}}^{2} \right)>\frac{\left( 1-r_{\mathrm{AX}}^{2} \right)^{2}}{\left( 1-r_{\mathrm{AB}}^{2} \right)}$$

$$1-\left( 1-r_{\mathrm{AB}}^{2} \right)<1-\frac{\left( 1-r_{\mathrm{AX}}^{2} \right)^{2}}{\left( 1-r_{\mathrm{AB}}^{2} \right)}$$

$$r_{\mathrm{AB}}^{2}<\frac{r_{\mathrm{BC}}^{2}-r_{E_{\mathrm{ba}}C}^{2}}{r_{\mathrm{BC}}^{2}}$$

**Pathway P5a: Single different cause**

The causal equations for this model would be

$$X=input=\bar{X}+e_{x}$$

$$A= input=\bar{A}+e_{a}$$

$$B= m_{1}A+m_{2}X+ e_{1}+ k_{1}$$

$$C= m_{3}X+ e_{2}+ k_{2}$$

where, $e_{x}$ ,$e_{a}, e_{1}$ and $e_{2}$ are not correlated.

The regression parameters can be derived from the causal equations as below

$$e_{x}=input$$

$$e_{a}=input$$

$$e_{b}=m_{1}e_{a}+m_{2}e_{x}+e_{1}$$

$$e_{c}=m_{3}e_{x}+e_{2}$$

$$M_{\mathrm{ba}}=\frac{\sum(e_{b})(e_{a})}{\sum e_{a}^{2}}=\frac{\sum(m_{2}e_{x}+m_{1}e_{a}+e_{2})e_{a}}{\sum e_{a}^{2}}=\frac{m_{2}\sum e_{x}.e_{a}}{\sum e_{a}^{2}}+\frac{m_{1}\sum e_{a}^{2}}{\sum e_{a}^{2}}+\frac{\sum e_{a}.e_{1}}{\sum e_{a}^{2}}=m_{1}$$

$$M_{\mathrm{cb}}=\frac{\sum e_{c}e_{b}}{\sum e_{b}^{2}}=\frac{\sum(m_{3}e_{x}+e_{2})(m_{1}e_{a}+m_{2}e_{x}+e_{1})}{\sum e_{b}^{2}}=\frac{m_{1}m_{3}\sum e_{x}.e_{a}}{\sum e_{b}^{2}}+\frac{m_{3}}{m_{2}}r_{\mathrm{BX}}^{2}=\frac{m_{3}}{m_{2}}r_{\mathrm{BX}}^{2}$$

$M_{\mathrm{ca}}=\frac{\sum e_{c}e_{a}}{\sum e_{a}^{2}}=0$ since there is no covariance between $e_{c}$ and $e_{a}$

$$E_{\mathrm{ba}}=e_{b}-M_{\mathrm{ba}}e_{a}=m_{2}e_{x}+m_{1}e_{a}+e_{1}-\left( m_{1}+\frac{m_{2}\sum e_{x}.e_{a}}{\sum e_{a}^{2}} \right)e_{a}=m_{2}e_{x}+e_{1}$$

$$E_{\mathrm{cb}}=e_{c}-M_{\mathrm{cb}}e_{b}=m_{3}e_{x}+e_{2}-\frac{m_{3}}{m_{2}}r_{\mathrm{BX}}^{2}\left( m_{1}e_{a}+m_{2}e_{x}+e_{1} \right)=m_{3}e_{x}+e_{2}-m_{3}r_{\mathrm{BX}}^{2}\left( \frac{m_{1}e_{a}}{m_{2}}+e_{x}+\frac{e_{1}}{m_{2}} \right)$$

$$E_{\mathrm{ca}}=e_{c}-M_{\mathrm{ca}}e_{a}=m_{3}e_{x}+e_{2}-m_{1}e_{a}$$

The relationships in table P5a in the main text are inferred from the above.

**Prediction R1**

$$r_{\mathrm{AB}}=\frac{\sum(e_{a})(e_{b})}{\sqrt{\sum e_{a}^{2}}\sqrt{\sum e_{b}^{2}}}=\frac{\sum(e_{a})(m_{1}e_{a}+m_{2}e_{x}+e_{1})}{\sqrt{\sum e_{a}^{2}}\sqrt{\sum e_{b}^{2}}}=\frac{m_{1}\sqrt{\sum e_{a}^{2}}}{\sqrt{\sum e_{b}^{2}}}$$

$$r_{\mathrm{BC}}=\frac{\sum(e_{c})(e_{b})}{\sqrt{\sum e_{c}^{2}}\sqrt{\sum e_{b}^{2}}}=\frac{\sum(m_{3}e_{x}+e_{2})(m_{1}e_{a}+m_{2}e_{x}+e_{1})}{\sqrt{\sum e_{c}^{2}}\sqrt{\sum e_{b}^{2}}}=\frac{m_{2}m_{3}\sum e_{x}^{2}}{\sqrt{\sum e_{c}^{2}}\sqrt{\sum e_{b}^{2}}}$$

$$r_{\mathrm{AC}}=\frac{\sum(e_{c})(e_{a})}{\sqrt{\sum e_{c}^{2}}\sqrt{\sum e_{a}^{2}}}=\frac{\sum(e_{a})(m_{3}e_{x}+e_{2})}{\sqrt{\sum e_{c}^{2}}\sqrt{\sum e_{a}^{2}}}=0$$

Since $r_{\mathrm{AB}}^{2}$ and $r_{\mathrm{BC}}^{2}$ are non zero positive,

$$r_{\mathrm{AC}}^{2}-r_{\mathrm{AB}}^{2}.r_{\mathrm{BC}}^{2}<0$$

**Prediction R2**

$$|M_{\mathrm{ca}}|-|M_{\mathrm{cb}}M_{\mathrm{ba}}|=|0|-|\frac{m_{3}}{m_{2}}r_{\mathrm{BX}}^{2}m_{1}|=-ve$$

**Prediction R3**

$$r_{E_{\mathrm{ba}}E_{\mathrm{cb}}}=\frac{\sum E_{\mathrm{ba}}E_{\mathrm{cb}}}{\sqrt{\sum E_{\mathrm{ba}}^{2}}\sqrt{\sum E_{\mathrm{cb}}^{2}}}=\frac{\sum\left( m_{2}e_{x}+e_{1} \right)\left( m_{3}e_{x}+e_{2}-\frac{{m_{1}m}_{3}}{m_{2}}r_{\mathrm{BX}}^{2}e_{a}-m_{3}e_{x}r_{\mathrm{BX}}^{2}-\frac{m_{3}}{m_{2}}r_{\mathrm{BX}}^{2}e_{1} \right)}{\sqrt{\sum E_{\mathrm{ba}}^{2}}\sqrt{\sum E_{\mathrm{cb}}^{2}}}=\frac{m_{2}m_{3}\sum e_{x}^{2}-m_{2}m_{3}\sum e_{x}^{2}r_{\mathrm{BX}}^{2}}{\sqrt{\sum E_{\mathrm{ba}}^{2}}\sqrt{\sum E_{\mathrm{cb}}^{2}}}=\frac{m_{2}m_{3}\sum e_{x}^{2}(1-r_{\mathrm{BX}}^{2})}{\sqrt{\sum E_{\mathrm{ba}}^{2}}\sqrt{\sum E_{\mathrm{cb}}^{2}}}=\frac{m_{2}m_{3}\sum e_{x}^{2}r_{\mathrm{AB}}^{2}}{\sqrt{\sum E_{\mathrm{ba}}^{2}}\sqrt{\sum E_{\mathrm{cb}}^{2}}}$$

which is postive when the signs of $m_{3}$ and $m_{4}$ are the same and negative when they are different.

**Prediction R4**

1. Since B which is correlated to A adds an error to $r_{\mathrm{BC}}$, a correction in A is expected to improve the correlation of B and C.

Formally,

$$r_{E_{\mathrm{ba}}C}=\frac{\sum(E_{\mathrm{ba}})(e_{c})}{\sqrt{\sum E_{\mathrm{ba}}^{2}}\sqrt{\sum e_{c}^{2}}}=\frac{\sum\left( m_{2}e_{x}+e_{1} \right)(m_{3}e_{x}+e_{2})}{\sqrt{\sum E_{\mathrm{ba}}^{2}}\sqrt{\sum e_{c}^{2}}}=\frac{m_{2}m_{3}\sum e_{x}^{2}}{\sqrt{\sum E_{\mathrm{ba}}^{2}}\sqrt{\sum e_{c}^{2}}}$$

$$r_{\mathrm{BC}}=\frac{\sum(e_{c})(e_{b})}{\sqrt{\sum e_{c}^{2}}\sqrt{\sum e_{b}^{2}}}=\frac{\sum(m_{3}e_{x}+e_{2})(m_{1}e_{a}+m_{2}e_{x}+e_{1})}{\sqrt{\sum e_{c}^{2}}\sqrt{\sum e_{b}^{2}}}=\frac{m_{2}m_{3}\sum e_{x}^{2}}{\sqrt{\sum e_{c}^{2}}\sqrt{\sum e_{b}^{2}}}$$

$$r_{\mathrm{BC}}=\frac{\sqrt{\sum E_{\mathrm{ba}}^{2}}}{\sqrt{\sum e_{b}^{2}}}r_{E_{\mathrm{ba}}C}$$

Since $\sqrt{\sum E_{\mathrm{ba}}^{2}}<\sqrt{\sum e_{b}^{2}}$

$$r_{\mathrm{BC}}<r_{E_{\mathrm{ba}}C}$$

1. Further $\frac{{r_{E_{\mathrm{ba}}C}^{2}-r}_{\mathrm{BC}}^{2}}{r_{E_{\mathrm{ba}}C}^{2}}=\frac{{r_{E_{\mathrm{ba}}C}^{2}-\frac{\sum E_{\mathrm{ba}}^{2}}{\sum e_{b}^{2}}r_{E_{\mathrm{ba}}C}}}{r_{E_{\mathrm{ba}}C}^{2}}=1-\frac{\sum E_{\mathrm{ba}}^{2}}{\sum e_{b}^{2}}$

= r^2^_AB_

Therefore $\frac{\left| r_{\mathrm{BC}}^{2} -r_{E_{\mathrm{ba}}C}^{2} \right|}{\max\left( r_{\mathrm{BC}}^{2}, r_{E_{\mathrm{ba}}C}^{2} \right)}=r_{\mathrm{AB}}^{2}$ is true.

**Pathway P5b: Double Different causes**

The causal equations for this model would be

$$X=input=\bar{X}+e_{x}$$

$$Y=input=\bar{Y}+e_{y}$$

$$A= m_{1}X+ e_{1}+ k_{1}$$

$$B= m_{2}X+m_{3}Y+ e_{2}+ k_{2}$$

$$C= m_{4}Y+ e_{3}+ k_{3}$$

where, $e_{x}$,$e_{y}$ ,$e_{1}$ , $e_{2}$ and $e_{3}$ are not correlated.

The regression parameters can be derived from the causal equations as below

$$e_{x}=input$$

$$e_{y}=input$$

$$e_{a}=m_{1}e_{x}+e_{1}$$

$$e_{b}=m_{2}e_{x}+m_{3}e_{y}+e_{2}$$

$$e_{c}=m_{4}e_{y}+e_{3}$$

$$M_{\mathrm{ba}}=\frac{\sum(e_{b})(e_{a})}{\sum e_{a}^{2}}=\frac{\sum(m_{2}e_{x}+m_{3}e_{y}+e_{2})(m_{1}e_{x}+e_{1})}{\sum e_{a}^{2}}=\frac{m_{1}m_{2}\sum e_{x}^{2}}{\sum e_{a}^{2}}=\frac{m_{2}}{m_{1}}\frac{m_{1}^{2}\sum e_{x}^{2}}{\sum e_{a}^{2}}=\frac{m_{2}}{m_{1}}r_{\mathrm{AX}}^{2}$$

$$M_{\mathrm{cb}}=\frac{\sum e_{c}e_{b}}{\sum e_{b}^{2}}=\frac{\sum(m_{4}e_{y}+e_{3})(m_{2}e_{x}+m_{3}e_{y}+e_{2})}{\sum e_{b}^{2}}=\frac{m_{4}m_{3}\sum e_{y}^{2}}{\sum e_{b}^{2}}=\frac{m_{4}}{m_{3}}\frac{m_{3}^{2}\sum e_{y}^{2}}{\sum e_{b}^{2}}=\frac{m_{4}}{m_{3}}r_{\mathrm{BY}}^{2}$$

$M_{\mathrm{ca}}=\frac{\sum e_{c}e_{a}}{\sum e_{a}^{2}}=0$ since there is no covariance between $e_{c}$ and $e_{a}$

$$E_{\mathrm{ba}}=e_{b}-M_{\mathrm{ba}}e_{a}=m_{2}e_{x}+m_{3}e_{y}+e_{2}-\frac{m_{2}}{m_{1}}r_{\mathrm{AX}}^{2}\left( m_{1}e_{x}+e_{1} \right)=m_{2}e_{x}\left( 1-r_{\mathrm{AX}}^{2} \right)+m_{3}e_{y}+e_{2}-\frac{m_{2}}{m_{1}}r_{\mathrm{AX}}^{2}e_{1}$$

$$E_{\mathrm{cb}}=e_{c}-M_{\mathrm{cb}}e_{b}=m_{4}e_{y}+e_{3}-\frac{m_{4}}{m_{3}}r_{\mathrm{BY}}^{2}\left( m_{2}e_{x}+m_{3}e_{y}+e_{2} \right)=m_{4}e_{y}\left( 1-r_{\mathrm{BY}}^{2} \right)+e_{3}-\frac{m_{4}}{m_{3}}r_{\mathrm{BY}}^{2}\left( m_{2}e_{x}+e_{2} \right)$$

$$E_{\mathrm{ca}}=e_{c}-M_{\mathrm{ca}}e_{a}=e_{c}-0=e_{c}=m_{4}e_{y}+e_{3}$$

The relationships in table P6 in the main text are inferred from the above.

**Prediction R1**

$$r_{\mathrm{AX}}=\frac{\sum(e_{a})(e_{x})}{\sqrt{\sum e_{a}^{2}}\sqrt{\sum e_{x}^{2}}}=\frac{\sum(m_{1}e_{x}+e_{1})e_{x}}{\sqrt{\sum e_{a}^{2}}\sqrt{\sum e_{x}^{2}}}=\frac{m_{1}\sum e_{x}^{2}}{\sqrt{\sum e_{a}^{2}}\sqrt{\sum e_{x}^{2}}}=\frac{m_{1}\sqrt{\sum e_{x}^{2}}}{\sqrt{\sum e_{a}^{2}}}$$

$$r_{BY}=\frac{m_{3}\sqrt{\sum e_{y}^{2}}}{\sqrt{\sum e_{b}^{2}}}$$

$$r_{AB}=\frac{\sum(e_{a})(e_{b})}{\sqrt{\sum e_{a}^{2}}\sqrt{\sum e_{b}^{2}}}=\frac{\sum(m_{1}e_{x}+e_{1})(m_{2}e_{x}+m_{3}e_{y}+e_{2})}{\sqrt{\sum e_{a}^{2}}\sqrt{\sum e_{b}^{2}}}=\frac{m_{1}m_{2}\sum e_{x}^{2}}{\sqrt{\sum e_{a}^{2}}\sqrt{\sum e_{b}^{2}}}$$

$$r_{BC}=\frac{\sum(e_{c})(e_{b})}{\sqrt{\sum e_{c}^{2}}\sqrt{\sum e_{b}^{2}}}=\frac{\sum(m_{4}e_{y}+e_{3})(m_{2}e_{x}+m_{3}e_{y}+e_{2})}{\sqrt{\sum e_{c}^{2}}\sqrt{\sum e_{b}^{2}}}=\frac{m_{3}m_{4}\sum e_{y}^{2}}{\sqrt{\sum e_{c}^{2}}\sqrt{\sum e_{b}^{2}}}$$

$$r_{AC}=\frac{\sum(e_{c})(e_{a})}{\sqrt{\sum e_{c}^{2}}\sqrt{\sum e_{a}^{2}}}=\frac{\sum(m_{4}e_{y}+e_{3})(m_{1}e_{x}+e_{1})}{\sqrt{\sum e_{c}^{2}}\sqrt{\sum e_{a}^{2}}}=0$$

Therefore as long as $r_{AB}$ and $r_{BC}$ are no zero,

$$r_{AC}^{2}-r_{AB}^{2}.r_{BC}^{2}<0$$

**Prediction R2**

$$|M_{ca}|-|M_{cb}M_{ba}|=|0|-|\frac{m_{4}}{m_{3}}r_{BY}^{2}\frac{m_{2}}{m_{1}}r_{AX}^{2}|=-ve$$

**Prediction R3**

$$r_{E_{ba}E_{cb}}=\frac{\sum E_{ba}E_{cb}}{\sqrt{\sum E_{ba}^{2}}\sqrt{\sum E_{cb}^{2}}}=\frac{\sum\left( m_{2}e_{x}\left( 1-r_{AX}^{2} \right)+m_{3}e_{y}+e_{2}-\frac{m_{2}}{m_{1}}r_{AX}^{2}e_{1} \right)\left( m_{4}e_{y}\left( 1-r_{BY}^{2} \right)+e_{3}-\frac{m_{4}}{m_{3}}r_{BY}^{2}\left( m_{2}e_{x}+e_{2} \right) \right)}{\sqrt{\sum E_{ba}^{2}}\sqrt{\sum E_{cb}^{2}}}$$

$$=\frac{-m_{2}^{2}\left( 1-r_{AX}^{2} \right)\frac{m_{4}}{m_{3}}r_{BY}^{2}\sum e_{x}^{2}+m_{3}m_{4}\left( 1-r_{BY}^{2} \right)\sum e_{y}^{2}-\frac{m_{4}}{m_{3}}r_{BY}^{2}\sum e_{2}^{2}}{\sqrt{\sum E_{ba}^{2}}\sqrt{\sum E_{cb}^{2}}}$$

$$=\frac{-m_{2}^{2}\left( 1-\frac{m_{1}^{2}\sum e_{x}^{2}}{\sum e_{a}^{2}} \right)\frac{m_{4}}{m_{3}}\frac{m_{3}^{2}\sum e_{y}^{2}}{\sum e_{b}^{2}}\sum e_{x}^{2}+m_{3}m_{4}\left( 1-\frac{m_{3}^{2}\sum e_{y}^{2}}{\sum e_{b}^{2}} \right)\sum e_{y}^{2}-\frac{m_{4}}{m_{3}}\frac{m_{3}^{2}\sum e_{y}^{2}}{\sum e_{b}^{2}}\sum e_{2}^{2}}{\sqrt{\sum E_{ba}^{2}}\sqrt{\sum E_{cb}^{2}}}$$

$$=\frac{-m_{2}^{2}m_{3}m_{4}\frac{\sum e_{1}^{2}}{\sum e_{a}^{2}}\frac{\sum e_{y}^{2}}{\sum e_{b}^{2}}\sum e_{x}^{2}+m_{3}m_{4}\left( \frac{m_{2}^{2}\sum e_{x}^{2}+\sum e_{2}^{2}}{\sum e_{b}^{2}} \right)\sum e_{y}^{2}-m_{3}m_{4}\frac{\sum e_{y}^{2}}{\sum e_{b}^{2}}\sum e_{2}^{2}}{\sqrt{\sum E_{ba}^{2}}\sqrt{\sum E_{cb}^{2}}}$$

$$=\frac{-m_{2}^{2}m_{3}m_{4}\frac{\sum e_{1}^{2}}{\sum e_{a}^{2}}\frac{\sum e_{y}^{2}}{\sum e_{b}^{2}}\sum e_{x}^{2}+m_{3}m_{4}\frac{m_{2}^{2}\sum e_{x}^{2}}{\sum e_{b}^{2}}\sum e_{y}^{2}}{\sqrt{\sum E_{ba}^{2}}\sqrt{\sum E_{cb}^{2}}}$$

$$=\frac{m_{2}^{2}m_{3}m_{4}\frac{\sum e_{y}^{2}}{\sum e_{b}^{2}}\sum e_{x}^{2}\left( -\frac{\sum e_{1}^{2}}{\sum e_{a}^{2}}+1 \right)}{\sqrt{\sum E_{ba}^{2}}\sqrt{\sum E_{cb}^{2}}}$$

$$=\frac{m_{2}^{2}m_{3}m_{4}\frac{\sum e_{y}^{2}}{\sum e_{b}^{2}}\sum e_{x}^{2}\left( r_{AX}^{2} \right)}{\sqrt{\sum E_{ba}^{2}}\sqrt{\sum E_{cb}^{2}}}$$

which is postive when the signs of $m_{3}$ and $m_{4}$ are the same and negative when they are different, so $r_{E_{ba}E_{cb}}$has the same sign as $M_{cb}$.

**Prediction R4**

1. Since X which is correlated to A adds to the error in $r_{BC}$ a correction for A is expected to improve the correlation of B with C.

Formally,

$$r_{E_{ba}C}=\frac{\sum(E_{ba})(e_{c})}{\sqrt{\sum E_{ba}^{2}}\sqrt{\sum e_{c}^{2}}}=\frac{\sum(m_{2}e_{x}\left( 1-r_{AX}^{2} \right)+m_{3}e_{y}+e_{2}-\frac{m_{2}}{m_{1}}r_{AX}^{2}e_{1})(m_{4}e_{y}+e_{3})}{\sqrt{\sum E_{ba}^{2}}\sqrt{\sum e_{c}^{2}}}=\frac{m_{3}m_{4}\sum e_{y}^{2}}{\sqrt{\sum E_{ba}^{2}}\sqrt{\sum e_{c}^{2}}}$$

$$r_{BC}=\frac{\sum(e_{c})(e_{b})}{\sqrt{\sum e_{c}^{2}}\sqrt{\sum e_{b}^{2}}}=\frac{\sum(m_{4}e_{y}+e_{3})(m_{2}e_{x}+m_{3}e_{y}+e_{2})}{\sqrt{\sum e_{c}^{2}}\sqrt{\sum e_{b}^{2}}}=\frac{m_{3}m_{4}\sum e_{y}^{2}}{\sqrt{\sum e_{c}^{2}}\sqrt{\sum e_{b}^{2}}}$$

$$r_{BC}=\frac{\sqrt{\sum E_{ba}^{2}}}{\sqrt{\sum e_{b}^{2}}}r_{E_{ba}C}$$

Since $\sqrt{\sum E_{ba}^{2}}<\sqrt{\sum e_{b}^{2}}$

$$r_{BC}<r_{E_{ba}C}$$

1. Further $\frac{{r_{E_{ba}C}^{2}-r}_{BC}^{2}}{r_{E_{ba}C}^{2}}=\frac{{r_{E_{ba}C}^{2}-\frac{\sum E_{ba}^{2}}{\sum e_{b}^{2}}r_{E_{ba}C}}}{r_{E_{ba}C}^{2}}=1-\frac{\sum E_{ba}^{2}}{\sum e_{b}^{2}}$

= r^2^_AB_

Therefore $\frac{\left| r_{BC}^{2} -r_{E_{ba}C}^{2} \right|}{max\left( r_{BC}^{2}, r_{E_{ba}C}^{2} \right)}=r_{AB}^{2}$ is true.

**Pathways with loops and cycles**

**Pathway P6: Negative or positive feedback loop**

$$A=input$$

$$\frac{dB}{dt}=m_{1}A-d_{1}B+e_{1}+m_{f}C+k_{1}$$

$$\frac{dC}{dt}=m_{2}B-d_{2}C+e_{2}+k_{2}$$

At equilibrium where both $\frac{dB}{dt}$ and $\frac{dC}{dt}$ =0, the equilibrium concentrations of B and C are given by

$$B=\frac{m_{1}d_{2}}{(d_{1}d_{2}-m_{2}m_{f})}A+\frac{d_{2}e_{1}+m_{f}e_{2}}{d_{1}d_{2}-m_{2}m_{f}}+\frac{m_{f}k_{2}+k_{1}d_{2}}{d_{1}d_{2}-m_{2}m_{f}}$$

$$C=\frac{m_{2}}{d_{2}}B+\frac{e_{2}}{d_{2}}+\frac{k_{2}}{d_{2}}$$

For simplification we take $m_{1}^{'}=\frac{m_{1}d_{2}}{(d_{1}d_{2}-m_{2}m_{f})}$ , $e_{1}^{'}=\frac{d_{2}e_{1}+m_{f}e_{2}}{d_{1}d_{2}-m_{2}m_{f}}$ and $k_{1}^{'}=\frac{m_{f}k_{2}+k_{1}d_{2}}{d_{1}d_{2}-m_{2}m_{f}}$

$B=m_{1}^{'}A+e_{1}^{'}$+$k_{1}^{'}$

Similarly $m_{2}^{'}=\frac{m_{2}}{d_{2}}$ , $e_{2}^{'}=\frac{e_{2}}{d_{2}}$ and $k_{2}^{'}=\frac{k_{2}}{d_{2}}$

$$C=m_{2}^{'}B+e_{2}^{'}+ k_{2}^{'}$$

It should be noted that $e_{1}^{'}=\frac{d_{2}e_{1}+m_{f}e_{2}}{d_{1}d_{2}-m_{2}m_{f}}$ and $e_{2}^{'}=\frac{e_{2}}{d_{2}}$ share $e_{2}$, therefore there will be a covariance between the two, the sign of which is decided by the sign of $m_{f}$, i.e. whether the feedback is positive or negative.

Regression parameters can be derived from the above as

$$e_{a}=input$$

$$e_{b}=m_{1}^{'}e_{a}+e_{1}^{'}$$

$$e_{c}=m_{2}^{'}e_{b}+e_{2}^{'}$$

$M_{ba}=\frac{\sum e_{a}e_{b}}{\sum e_{a}^{2}}=\frac{\sum e_{a}(m_{1}^{'}e_{a}+e_{1}^{`})}{\sum e_{a}^{2}}=\frac{m_{1}^{`}\sum e_{a}^{2}}{\sum e_{a}^{2}}=m_{1}^{`}$=$\frac{m_{1}d_{2}}{(d_{1}d_{2}-m_{2}m_{f})}$

$M_{cb}=\frac{\sum e_{c}e_{b}}{\sum e_{b}^{2}}=\frac{\sum(m_{2}^{`}e_{b}+e_{2}^{`})(m_{1}^{`}e_{a}+e_{1}^{`})}{\sum e_{b}^{2}}=\frac{m_{2}^{`}\sum e_{b}^{2}+\sum e_{1}^{`}e_{2}^{`}}{\sum e_{b}^{2}}=m_{2}^{`}+\frac{\sum e_{1}^{`}e_{2}^{`}}{\sum e_{b}^{2}}$ =$\frac{m_{2}}{d_{2}}+\frac{\sum e_{1}^{`}e_{2}^{`}}{\sum e_{b}^{2}}$

$$M_{ca}=\frac{\sum e_{c}e_{a}}{\sum e_{a}^{2}}=\frac{\sum(m_{2}^{`}m_{1}^{`}e_{a}+m_{2}^{`}e_{1}^{`}+e_{2}^{`})e_{a}}{\sum e_{a}^{2}}=\frac{m_{2}^{`}m_{1}^{`}\sum e_{a}^{2}}{\sum e_{a}^{2}}=\frac{m_{2}}{d_{2}}\frac{m_{1}d_{2}}{(d_{1}d_{2}-m_{2}m_{f})}$$

$$E_{ba}=e_{b}-M_{ba}e_{a}=\left( m_{1}^{`}e_{a}+e_{1}^{`} \right)-m_{1}^{`}\left( e_{a} \right)=e_{1}^{`}=\frac{m_{f}e_{2}+d_{2}e_{1}}{(d_{1}d_{2}-m_{2}m_{f})}$$

$$E_{cb}=e_{c}-M_{cb}e_{b}=\left( m_{2}^{`}e_{b}+e_{2}^{`} \right)-\left( m_{2}^{`}+\frac{\sum e_{1}^{`}e_{2}^{`}}{\sum e_{b}^{2}} \right)\left( e_{b} \right)=\frac{e_{2}}{d_{2}}-\frac{Cov (\frac{d_{2}e_{1}+m_{f}e_{2}}{d_{1}d_{2}-m_{2}m_{f}})(\frac{e_{2}}{d_{2}})}{\sum e_{b}^{2}}e_{b}$$

$$E_{ca}=e_{c}-M_{ca}e_{a}=\left( m_{2}^{`}m_{1}^{`}e_{a}+m_{2}^{`}e_{1}^{`}+e_{2}^{`} \right)-m_{2}^{`}m_{1}^{`}\left( e_{a} \right)=\frac{m_{2}}{d_{2}}\frac{d_{2}e_{1}+m_{f}e_{2}}{d_{1}d_{2}-m_{2}m_{f}}+\frac{e_{2}}{d_{2}}$$

The relationships in table P6 in the main text are inferred from the above.

**Prediction R1**

In the general equation 1 since there is covariance between $E_{ba}$ and $E_{cb}$ in the feedback pathways, and $M_{ca}$ is not predicted by $M_{ba}$ and $M_{cb}$, $r_{AC}^{2} is not predicted by the product r_{AB}^{2}.r_{BC}^{2}$.For negative feedbacks $M_{ca}^{2}>M_{ba}^{2}.M_{cb}^{2}$ and the covarience term is negative, therefore

$\frac{M_{ba}^{2}.M_{cb}^{2}.\sigma^{2}A}{M_{ba}^{2}.M_{cb}^{2}.\sigma^{2}A+M_{cb}^{2}\sigma^{2}E_{ba}+\sigma^{2}E_{cb}}$ < $\frac{M_{ca}^{2}.\sigma^{2}A}{M_{ca}^{2}.\sigma^{2}A+\sigma^{2}E_{cA}}$

$r_{AC}^{2}>r_{AB}^{2}.r_{BC}^{2}$

The reverse applies for positive feedbacks where $M_{ca}^{2}<M_{ba}^{2}.M_{cb}^{2}$ and the covariance term is positive, therefore

$\frac{M_{ba}^{2}.M_{cb}^{2}.\sigma^{2}A}{M_{ba}^{2}.M_{cb}^{2}.\sigma^{2}A+M_{cb}^{2}\sigma^{2}E_{ba}+\sigma^{2}E_{cb}}$ >

$r_{AC}^{2}<r_{AB}^{2}.r_{BC}^{2}$

**Prediction R2**

From table P6

$M_{ba}=m_{1}^{`}$, $M_{cb}=m_{2}^{`}+\frac{\sum e_{1}^{`}e_{2}^{`}}{\sum e_{b}^{2}}$ and $M_{ca}=m_{2}^{`}m_{1}^{`}$

Therefore, $M_{ca}=M_{ba}{(M}_{cb}$-$\frac{\sum e_{1}^{`}e_{2}^{`}}{\sum e_{b}^{2}})$

Since the covariance $e_{1}^{'}e_{2}^{'}$ is negative in negative feedback and when it is positive i.e. in positive feedback .

**Prediction R3**

From table P6:

$$r_{E_{ba}E_{cb}}=\frac{\sum E_{ba}E_{cb}}{\sqrt{\sum E_{ba}^{2}}\sqrt{\sum E_{cb}^{2}}}=\frac{\sum\left( e_{1}^{`} \right)\left( e_{2}^{`}-\frac{\sum e_{1}^{`}e_{2}^{`}}{\sum e_{b}^{2}}e_{b} \right)}{\sqrt{\sum E_{ba}^{2}}\sqrt{\sum E_{cb}^{2}}}$$

Substituting e’_1_ and e’_2_ and simplifying we get

$$=\frac{m_{f}}{d_{2}\left( d_{1}d_{2}-m_{2}m_{f} \right)}\sum e_{2}^{2}\left( 1-\frac{\sum e_{1}^{`2}}{\sum e_{b}^{2}} \right)$$

Since $e_{b}=m_{1}^{'}e_{a}+e_{1}^{'}$

so $\sum e_{b}^{2}>\sum e_{1}^{`2}$ and the bracket term will be positive. In the case of negative feedback, the denominator will be positive and therefore the sign of this correlation will be decided by the sign of $m_{f}$ which is negative. For positive feedback since $d_{1}d_{2}>m_{2}m_{f}$ for a positive steady state B, and since $m_{f}$ is positive the correlation will be positive.

**Prediction R4**

$r_{E_{ba}C}=\frac{\sum(E_{ba})(e_{c})}{\sqrt{\sum E_{ba}^{2}}\sqrt{\sum e_{c}^{2}}}=\frac{\sum(e_{1}^{`})(m_{2}^{`}m_{1}^{`}e_{a}+m_{2}^{`}e_{1}^{`}+e_{2}^{`})}{\sqrt{\sum e_{1}^{`2}}\sqrt{\sum e_{c}^{2}}}=\frac{m_{2}^{`}\sum e_{1}^{`2}+\sum e_{1}^{'}e_{2}^{'}}{\sqrt{\sum e_{1}^{`2}}\sqrt{\sum e_{c}^{2}}}$

$$r_{BC}=\frac{m_{2}^{'}\sum e_{b}^{2}+\sum e_{1}^{'}e_{2}^{'}}{\sqrt{\sum e_{b}^{2}}\sqrt{\sum e_{c}^{2}}}$$

$$r_{E_{ba}C}=\frac{m_{2}^{`}\sum e_{1}^{`2}+\sum e_{1}^{'}e_{2}^{'}}{\sqrt{\sum e_{1}^{`2}}\sqrt{\sum e_{c}^{2}}}$$

$$r_{E_{ba}C}=\frac{\sqrt{\sum e_{b}^{2}}}{\sqrt{\sum e_{1}^{`2}}}\frac{m_{2}^{`}\sum e_{1}^{`2}+\sum e_{1}^{'}e_{2}^{'}}{m_{2}^{'}\sum e_{b}^{2}+\sum e_{1}^{'}e_{2}^{'}}\left( r_{BC} \right)$$

$$r_{E_{ba}C}=\frac{\frac{\sqrt{\sum e_{1}^{`2}}}{\sqrt{\sum e_{b}^{2}}}+\frac{\sum e_{1}^{'}e_{2}^{'}}{m_{2}^{`}\sqrt{\sum e_{1}^{`2}}\sqrt{\sum e_{b}^{2}}}}{1+\frac{\sum e_{1}^{'}e_{2}^{'}}{m_{2}^{`}\sqrt{\sum e_{b}^{2}}\sqrt{\sum e_{b}^{2}}}}\left( r_{BC} \right)$$

For negative feedbacks covariance between $e_{1}$ and $e_{2}$ is negative. In addition since $\sum e_{1}^{`2}$ is smaller than $\sum e_{b}^{2}$ the numerator has a smaller positive and larger negative term and the denominator has a larger positive and smaller negative term. Therefore, $r_{E_{ba}C}<\left( r_{BC} \right)$. For positive feedback the prediction is more conditional. Since all terms are positive the prediction depends upon their relative magnitude. Since covariance between $e_{1}$ and $e_{2}$ is mainly decided by $e_{2}$ it can be ascertained that unless $e_{2}$ is substantially large the second term will be much smaller than the first in both the numerator and denominator. As $e_{2}$ mainly decides $r_{BC}$ , $r_{E_{ba}C}<\left( r_{BC} \right)$ will be true above a threshold $r_{BC}$. For smaller $r_{BC}$ it is difficult to make a definite prediction. Simulations support this prediction.

1. Since

$$r_{E_{ba}C}=\frac{\frac{\sqrt{\sum e_{1}^{`2}}}{\sqrt{\sum e_{b}^{2}}}+\frac{\sum e_{1}^{'}e_{2}^{'}}{m_{2}^{`}\sqrt{\sum e_{1}^{`2}}\sqrt{\sum e_{b}^{2}}}}{1+\frac{\sum e_{1}^{'}e_{2}^{'}}{m_{2}^{`}\sqrt{\sum e_{b}^{2}}\sqrt{\sum e_{b}^{2}}}}\left( r_{BC} \right)$$

In a limiting case when covariance between $e_{1}$ and $e_{2}$ is very small,

$$r_{E_{baC}}^{2}=\frac{\sum e_{1}^{`2}}{\sum e_{b}^{2}}r_{BC}^{2}$$

Therefore

$\frac{r_{BC}^{2}-r_{E_{ba}C}^{2}}{r_{BC}^{2}}=1-$

$\frac{\sum e_{1}^{`2}}{\sum e_{b}^{2}}$

And since from table P6, $E_{ba}=e_{1}^{'}$

$\frac{r_{BC}^{2}-r_{E_{ba}C}^{2}}{r_{BC}^{2}}=1-$

$\frac{\sum E_{ba}^{2}}{\sum e_{b}^{2}}=r_{AB}^{2}$

However in reality, because of the covariance term, $r_{E_{ba}C}$ is further reduced, that means the percentage difference is further increased for negative feedback

$r_{E_{baC}}^{2}<\frac{e_{1}^{`2}}{e_{b}^{2}}r_{BC}^{2}$ Therefore $\frac{r_{BC}^{2}-r_{E_{ba}C}^{2}}{r_{BC}^{2}}>r_{AB}^{2}$

And for positive feedback, this time the covariance term being positive increases $r_{E_{ba}C}$, and so the percentage diff is then reduced, so

$r_{E_{baC}}^{2}>\frac{e_{1}^{`2}}{e_{b}^{2}}r_{BC}^{2}$ Therefore $\frac{r_{BC}^{2}-r_{E_{ba}C}^{2}}{r_{BC}^{2}}<r_{AB}^{2}$

**Pathway P7: Positive or negative feedforward**

Assumed model of causation;

$A=input= \bar{\bar{A}}+e_{a}$

$\frac{dB}{dt}= m_{1}A-d_{1}B+e_{1}+k_{1}=0$

$\frac{dC}{dt}= m_{2}B+m_{3}A-d_{2}C+e_{2}+k_{2}=0$

where ,$e_{a}$ $e_{1}$ and $e_{2}$ are Not correlated. At equilibrium

$B=\frac{m_{1}}{d_{1}}A+\frac{e_{1}}{d_{1}}+\frac{k_{1}}{d_{1}}=m_{1}^{`}A+e_{1}^{`}+k_{1}^{`}$ taking $m_{1}^{`}=\frac{m_{1}}{d_{1}}$ , $e_{1}^{`}=\frac{e_{1}}{d_{1}}$ and , $k_{1}^{`}=\frac{k_{1}}{d_{1}}$

$$C=\frac{m_{1}m_{2}+d_{1}m_{f}}{d_{1}d_{2}}A+(\frac{m_{2}}{d_{1}d_{2}}e_{1}+\frac{1}{d_{2}}e_{2})$$

$$C=\frac{m_{1}m_{2}+m_{f}d_{1}}{d_{2}m_{1}}B-\frac{m_{f}}{m_{1}d_{2}}e_{1}+\frac{e_{2}}{d_{2}}+\frac{k_{2}}{d_{2}}$$

We will take $m_{2}^{`}=\frac{m_{1}m_{2}+m_{f}d_{1}}{d_{2}m_{1}} and e_{2}^{`}=$ $-\frac{m_{f}}{m_{1}d_{2}}e_{1}+\frac{e_{2}}{d_{2}}$

Note that since e1 decides both $e_{1}^{`}$and $e_{2}^{`}$, the covariance between $e_{1}^{`}$ and $e_{2}^{`}$ will be

$-\frac{m_{f}}{m_{1}d_{2}}e_{1}$ which will be positive when mf is negative i.e. for negative feedforward and negative when $m_{f}$ is positive i.e. positive feed forward.

Regression parameters can be derived as

$$e_{a}=input$$

$$e_{b}=m_{1}^{'}e_{a}+e_{1}^{'}$$

$$e_{c}=m_{2}^{'}e_{b}+e_{2}^{'}$$

$$r_{AB}=\frac{\sum e_{a}e_{b}}{\sqrt{\sum e_{a}^{2}}\sqrt{\sum e_{b}^{2}}}=\frac{\sum e_{a}(m_{1}^{'}e_{a}+e_{1}^{'})}{\sqrt{\sum e_{a}^{2}}\sqrt{\sum e_{b}^{2}}}=\frac{m_{1}^{'}\sum e_{a}^{2}}{\sqrt{\sum e_{a}^{2}}\sqrt{\sum e_{b}^{2}}}=m_{1}^{'}\frac{\sqrt{\sum e_{a}^{2}}}{\sqrt{\sum e_{b}^{2}}}$$

$$r_{BC}=\frac{\sum(e_{b})(e_{c})}{\sqrt{\sum e_{b}^{2}}\sqrt{\sum e_{c}^{2}}}=\frac{\sum(m_{2}^{'}e_{b}+e_{2}^{'})(m_{1}^{'}e_{a}+e_{1}^{'})}{\sqrt{\sum e_{b}^{2}}\sqrt{\sum e_{c}^{2}}}=\frac{m_{2}^{'}\sum e_{b}^{2}+\sum e_{1}^{'}e_{2}^{'}}{\sqrt{\sum e_{b}^{2}}\sqrt{\sum e_{c}^{2}}}=\frac{m_{2}^{'}\sqrt{\sum e_{b}^{2}}}{\sqrt{\sum e_{c}^{2}}}+\frac{\sum e_{1}^{'}e_{2}^{'}}{\sqrt{\sum e_{b}^{2}}\sqrt{\sum e_{c}^{2}}}$$

$$r_{AC}=\frac{\sum(e_{a})(e_{c})}{\sqrt{\sum e_{a}^{2}}\sqrt{\sum e_{c}^{2}}}=\frac{\sum(m_{2}^{`}m_{1}^{`}e_{a}+m_{2}^{`}e_{1}^{`}+e_{2}^{`})e_{a}}{\sqrt{\sum e_{a}^{2}}\sqrt{\sum e_{c}^{2}}}=\frac{m_{2}^{`}m_{1}^{`}\sum e_{a}^{2}}{\sqrt{\sum e_{a}^{2}}\sqrt{\sum e_{c}^{2}}}=m_{2}^{`}m_{1}^{`}\frac{\sqrt{\sum e_{a}^{2}}}{\sqrt{\sum e_{c}^{2}}}$$

$$M_{ba}=\frac{\sum e_{a}e_{b}}{\sum e_{a}^{2}}=\frac{\sum e_{a}(m_{1}^{`}e_{a}+e_{1}^{`})}{\sum e_{a}^{2}}=\frac{m_{1}^{`}\sum e_{a}^{2}}{\sum e_{a}^{2}}=m_{1}^{`}$$

$$M_{cb}=\frac{\sum e_{c}e_{b}}{\sum e_{b}^{2}}=\frac{\sum(m_{2}^{`}e_{b}+e_{2}^{`})(m_{1}^{`}e_{a}+e_{1}^{`})}{\sum e_{b}^{2}}=\frac{m_{2}^{`}\sum e_{b}^{2}+\sum e_{1}^{`}e_{2}^{`}}{\sum e_{b}^{2}}=m_{2}^{`}+\frac{\sum e_{1}^{`}e_{2}^{`}}{\sum e_{b}^{2}}$$

$$M_{ca}=\frac{\sum e_{c}e_{a}}{\sum e_{a}^{2}}=\frac{\sum(m_{2}^{`}m_{1}^{`}e_{a}+m_{2}^{`}e_{1}^{`}+e_{2}^{`})e_{a}}{\sum e_{a}^{2}}=\frac{m_{2}^{`}m_{1}^{`}\sum e_{a}^{2}}{\sum e_{a}^{2}}=m_{2}^{`}m_{1}^{`}$$

$$E_{ba}=e_{b}-M_{ba}e_{a}=\left( m_{1}^{`}e_{a}+e_{1}^{`} \right)-m_{1}^{`}\left( e_{a} \right)=e_{1}^{`}=\frac{m_{2}e_{2}+d_{2}e_{1}}{(d_{1}d_{2}-m_{2}m_{f})}$$

$$E_{cb}=e_{c}-M_{cb}e_{b}=\left( m_{2}^{`}e_{b}+e_{2}^{`} \right)-\left( m_{2}^{`}+\frac{\sum e_{1}^{`}e_{2}^{`}}{\sum e_{b}^{2}} \right)\left( e_{b} \right)=e_{2}^{`}-\frac{\sum e_{1}^{`}e_{2}^{`}}{\sum e_{b}^{2}}e_{b}$$

$$E_{ca}=e_{c}-M_{ca}e_{a}=\left( m_{2}^{`}m_{1}^{`}e_{a}+m_{2}^{`}e_{1}^{`}+e_{2}^{`} \right)-m_{2}^{`}m_{1}^{`}\left( e_{a} \right)=m_{2}^{`}e_{1}^{`}+e_{2}^{`}$$

The relationships in table P7 in the main text are inferred from the above.

**Prediction R1**

In the general equation 1 since there is covariance between $E_{ba}$ and $E_{cb}$ in the feedback pathways, and $M_{ca}$ is not predicted by $M_{ba}$ and M_cb_, $r_{AC}^{2}$ is not predicted by the product $r_{AB}^{2}r_{BC}^{2}$. For positive feedforward $M_{ca}^{2}>M_{ba}^{2}.M_{cb}^{2}$ and the covarience term is negative, therefore

$\frac{M_{ba}^{2}.M_{cb}^{2}.\sigma^{2}A}{M_{ba}^{2}.M_{cb}^{2}.\sigma^{2}A+M_{cb}^{2}\sigma^{2}E_{ba}+\sigma^{2}E_{cb}}$ < $\frac{M_{ca}^{2}.\sigma^{2}A}{M_{ca}^{2}.\sigma^{2}A+\sigma^{2}E_{cA}}$

$$r_{AC}^{2}>r_{AB}^{2}.r_{BC}^{2}$$

In the case of negative feedforward if $m_{1}m_{2}>d_{1}m_{f}$, then $M_{ca}^{2}<M_{ba}^{2}.M_{cb}^{2}$ and the covariance term is positive, therefore

$\frac{M_{ba}^{2}.M_{cb}^{2}.\sigma^{2}A}{M_{ba}^{2}.M_{cb}^{2}.\sigma^{2}A+M_{cb}^{2}\sigma^{2}E_{ba}+\sigma^{2}E_{cb}}$ >

$$r_{AC}^{2}<r_{AB}^{2}.r_{BC}^{2}$$

But if but if the prediction mimics positive feedforward since $M_{CA}^{2}>M_{BA}^{2}.M_{CB}^{2}$ therefore

$\frac{M_{BA}^{2}.M_{CB}^{2}.\sigma^{2}A}{M_{BA}^{2}.M_{CB}^{2}.\sigma^{2}A+M_{CB}^{2}\sigma^{2}E_{ba}+\sigma^{2}E_{cb}}$ < $\frac{M_{CA}^{2}.\sigma^{2}A}{M_{CA}^{2}.\sigma^{2}A+\sigma^{2}E_{cA}}$

$$r_{AC}^{2}>r_{AB}^{2}.r_{BC}^{2}$$

This is the condition when causally negative feedforward transforms into an effective positive feedforward and the prediction of positive feedforward is true.

**Prediction R2**

$$M_{cb}M_{ba}=m_{1}^{`}\left( m_{2}^{`}+\frac{\sum e_{1}^{`}e_{2}^{`}}{\sum e_{b}^{2}} \right)$$

Where, $m_{2}^{'}=\frac{m_{1}m_{2}+d_{1}m_{f}}{m_{1}d_{2}}$ , $\frac{\sum e_{1}^{`}e_{2}^{`}}{\sum e_{b}^{2}}=-\frac{m_{f}}{m_{1}d_{2}}\frac{\sum e_{1}^{2}}{\sum e_{b}^{2}}$

When mf is positive the covariance term is negative, therefore

$$M_{cb}M_{ba}<m_{1}^{`}m_{2}^{`}= M_{ca}$$

So, $|M_{ca}|-|M_{cb}M_{ba}|=+ve$

In the case of negative feedforward since mf is negative the covariance term is positive. If $m_{2}^{'}$ is positive i.e. $m_{1}m_{2}>d_{1}m_{f}$ then

$M_{cb}M_{ba}>m_{1}^{`}m_{2}^{`}= M_{ca}$ or $|M_{ca}|-|M_{cb}M_{ba}|=-ve$

but if such that then $M_{cb}M_{ba}<m_{1}^{`}m_{2}^{`}= M_{ca}$ and, $|M_{ca}|-|M_{cb}M_{ba}|=+ve$. Note that this is the condition under which a negative feedforward transforms itself into a positive feedforward and the prediction of positive feedforward is true.

**Prediction R3**

Since $E_{ba}=e_{1}^{`}=\frac{e_{1}}{d_{1}}$ and

$$E_{cb}=e_{2}^{`}-\frac{\sum e_{1}^{`}e_{2}^{`}}{\sum e_{b}^{2}}e_{b}=-\frac{m_{f}}{m_{1}d_{2}}e_{1}+\frac{e_{2}}{d_{2}}-\frac{\sum e_{1}^{`}e_{2}^{`}}{\sum e_{b}^{2}}e_{b}$$

The sign of $m_{f}$ and $\sum e_{1}^{`}e_{2}^{`}$ decides the sign of correlation between $E_{ba}$ and $E_{cb}$. For positive feedback since both are positive we expect a negative correlation and for negative feedback both being negative will give rise to a positive correlation. Since this prediction does not depend upon the relative magnitudes of any of the parameters it helps us to distinguish between a causally positive feedforward and a causally negative feedforward turning into an effectively positive feedforward. For the former $M_{ca}$ is positive but $r_{E_{ba}E_{cb}}$ is negative but for the latter $M_{ca}$ is negative and $r_{E_{ba}E_{cb}}$ is positive.

**Prediction R4**

1. Similar to the feedback pathway

$$r_{E_{ba}C}=\frac{\frac{\sqrt{\sum e_{1}^{`2}}}{\sqrt{\sum e_{b}^{2}}}+\frac{\sum e_{1}^{'}e_{2}^{'}}{m_{2}^{`}\sqrt{\sum e_{1}^{`2}}\sqrt{\sum e_{b}^{2}}}}{1+\frac{\sum e_{1}^{'}e_{2}^{'}}{m_{2}^{`}\sqrt{\sum e_{b}^{2}}\sqrt{\sum e_{b}^{2}}}}\left( r_{BC} \right)$$

as $e_{b}=m_{1}^{'}e_{a}+e_{1}^{'}$

so $\sum e_{b}^{2}>\sum e_{1}^{`2}$

In the case of positive feedforward since $m_{f}$ as well as the covariance term are positive

the second terms in both the numerator and denominator are negative. Since the numerator is a smaller number subtracted by bigger number

while the denominator is a bigger number subtracted by a smaller number. Therefore, $r_{E_{ba}C}<r_{BC}$.

In the case of negative feedforward $m_{f}$ and the covariance term are negative making the prediction conditional. $\frac{\sum e_{1}^{`}e_{2}^{`}}{\sum e_{b}^{2}}=-\frac{m_{f}}{m_{1}d_{2}}\frac{\sum e_{1}^{2}}{\sum e_{b}^{2}}$, Since covariance between $e_{1}^{`}$ and $e_{2}^{`}$ is mainly decided by $e_{1}$ it can be ascertained that unless $e_{1}$ is substantially large the second term will be much smaller than the first in both the numerator and denominator. As $e_{1}$ mainly decides $r_{AB}$ , $r_{E_{ba}C}<\left( r_{BC} \right)$ will be true above a threshold r_AB_. For smaller $r_{AB}$ it is difficult to make a definite prediction.

1. Similar to feedback pathway

$$r_{E_{ba}C}=\frac{\frac{\sqrt{\sum e_{1}^{`2}}}{\sqrt{\sum e_{b}^{2}}}+\frac{\sum e_{1}^{'}e_{2}^{'}}{m_{2}^{`}\sqrt{\sum e_{1}^{`2}}\sqrt{\sum e_{b}^{2}}}}{1+\frac{\sum e_{1}^{'}e_{2}^{'}}{m_{2}^{`}\sqrt{\sum e_{b}^{2}}\sqrt{\sum e_{b}^{2}}}}\left( r_{BC} \right)$$

In a limiting case when covariance between $e_{1}^{`}$ and $e_{2}^{`}$ is very small,

$$r_{E_{ba}C}^{2}=\frac{\sum e_{1}^{`2}}{\sum e_{b}^{2}}r_{BC}^{2}$$

Therefore

$\frac{r_{BC}^{2}-r_{E_{ba}C}^{2}}{r_{BC}^{2}}=1-$

$\frac{\sum e_{1}^{`2}}{\sum e_{b}^{2}}$

And since from table P6, E_ba_ = e_1_’

$\frac{r_{BC}^{2}-r_{E_{ba}C}^{2}}{r_{BC}^{2}}=1-$

$\frac{\sum E_{ba}^{2}}{\sum e_{b}^{2}}=r_{AB}^{2}$

However in reality, for positive feedforward, because of the covariance term, $r_{E_{ba}C}$ is further reduced, that means the percentage difference is further increased,

$r_{E_{baC}}^{2}<\frac{e_{1}^{`2}}{e_{b}^{2}}r_{BC}^{2}$ Therefore $\frac{r_{BC}^{2}-r_{E_{ba}C}^{2}}{r_{BC}^{2}}>r_{AB}^{2}$

And for negative feedforward, this time the covariance term being positive increases $r_{E_{ba}C}$, and so the percentage diff is then reduced, so

$r_{E_{baC}}^{2}>\frac{e_{1}^{`2}}{e_{b}^{2}}r_{BC}^{2}$ Therefore $\frac{r_{BC}^{2}-r_{E_{ba}C}^{2}}{r_{BC}^{2}}<r_{AB}^{2}$

However, in reality,

$r_{E_{baC}}^{2}<\frac{e_{1}^{`2}}{e_{b}^{2}}r_{BC}^{2}$ Therefore $\frac{r_{BC}^{2}-r_{E_{ba}C}^{2}}{r_{BC}^{2}}>r_{AB}^{2}$

For negative feedforward, the opposite prediction to positive feedforward was in place had there not been an added complication, that is the sign of $m_{2}^{'}{; m}_{2}^{'}=\frac{m_{1}m_{2}+d_{1}m_{f}}{m_{1}d_{2}}$ , a universal prediction cannot be made, as now

$r_{E_{baC}}^{2}\leq>\frac{e_{1}^{`2}}{e_{b}^{2}}r_{BC}^{2}$ Therefore $\frac{r_{BC}^{2}-r_{E_{ba}C}^{2}}{r_{BC}^{2}}\leq>r_{AB}^{2}$.

**Calculation of causal parameters from regression parameters**

Since the regression correlation parameters are obtained from data, assuming a specific pathway it is possible to calculate the parameters of causal equations from regression equations for most of the pathways (see below). For some of the pathways the number of causal parameters are greater than the number of equations, in which case regression equations are not sufficient to work back causal equations. However, in glucose homeostasis, empirical estimates are available for at least some of the causal parameters. Therefore it was possible to obtain causal parameters for every putative causal pathway and using these parameters for Monte-Carlo simulations.

**Calculations of pathway specific causal parameters**

**P1: Linear Pathway**

$e_{1}= E_{ba}$

$e_{2}= E_{cb}$

$m_{1}= M_{ba}$

$m_{2}= M_{cb}$

**P2:Radiating Pathway**

1. $M_{ba}=\frac{1}{m_{1}}r_{AB}^{2} Therefore, m_{1}= \frac{r_{AB}^{2}}{M_{ba}}$ …. Equation 1

$m_{2}=M_{cb}$

$e_{2}=E_{cb}$

2. $E_{ba}=e_{b}(1-r_{AB}^{2})-\frac{1}{m_{1}}r_{AB}^{2}e_{1}$ Substituting equation 1 here,

$$e_{1}=\frac{e_{b}-E_{ba}-e_{b}.r_{AB}^{2}}{M_{ba}}$$

**P3: Converging Pathway**

$m_{1}=M_{ba}$

1. $M_{cb}=\frac{1}{m_{2}}r_{BC}^{2} Therefore, m_{2}= \frac{r_{BC}^{2}}{M_{cb}}$ …. Equation 2
2. $E_{ba}=e_{1}+m_{2}e_{c} Therefore, e_{1}=E_{ba}-m_{2}e_{c}$ Substituting equation 2 here,

$$e_{1}=E_{ba}-\frac{r_{BC}^{2}}{M_{cb}}e_{c}$$

**P4: Single Different Cause Pathway**

$m_{1}=M_{ba}$

To calculate the remaining causal parameters, we need to simulate X. We calculated the regression parameters viz. slopes M_ba_, M_ca_ and M_cb_; and intercepts K_ba_, K_ca_ and K_cb_ from the dataset. Using these we calculated the errors E_ba_, E_cb_ and E_ca_ using –

$E_{ba}=B-\left( M_{ba}.A+K_{ba} \right), E_{cb}=C-(M_{cb}.B+K_{cb})$, $E_{ca}=C-(M_{ca}.A+K_{ca})$

In case of a radiating pathway where X causes B and C,

$M_{cb}=M_{cx}.M_{xb}$ Assuming M_xb_ = 1, $M_{cb}=M_{cx}$

Hence, the maximum variance that can be obtained in the error E_xb_ is variance in the error E_cb_.

Therefore, we randomized σ^2^E_xb_ from 0 to σ^2^E_cb_. We then generated a list of respective standard deviations. We used 0 as mean and the obtained standard deviation to generate random value (E_xb_) from the distribution. We then generated a series of X corresponding to the series of B using –

$$X={(B*M}_{xb})+E_{xb}$$

We used this series of X as data and calculated the regression parameters E_cx_, E_bx_, M_bx_, M_cx_, K_bx_ and K_cx_. The standard deviation and mean of the X series was taken as input for the further Monte Carlo Simulations.

**P5: Negative Feedback Pathway**

The relationships between the regression and causal parameters is listed in Table 3. We derived equations for causal parameters from these relationships.

Table 3: Relationships between regression and causal parameters for the negative feedback pathway.

From equation 4,

${m_{1}=M}_{ca}\frac{(d_{1}d_{2}-m_{2}m_{f})}{m_{2}}$ …. Equation 5

Substituting equation 5 in equation 3,

$M_{ba}=\frac{M_{ca}\frac{(d_{1}d_{2}-m_{2}m_{f})}{m_{2}}d_{2}}{(d_{1}d_{2}-m_{2}m_{f})}=\frac{M_{ca}d_{2}}{m_{2}} Therefore, m_{2}=\frac{M_{ca}d_{2}}{M_{ba}}$

Substituting this equation for m_2_ in equation 5,

${m_{1}=M}_{ca}\frac{(d_{1}d_{2}-\frac{M_{ca}d_{2}}{M_{ba}}m_{f})}{\frac{M_{ca}d_{2}}{M_{ba}}}$=$d_{1}M_{ba}-m_{f}M_{ca}$

From equations 6 and 7,

$$E_{ca}=\frac{m_{2}}{d_{2}}E_{ba}+\frac{e_{2}}{d_{2}}Therefore, E_{ca}d_{2}=m_{2}E_{ba}+e_{2}$$

Substituting this equation for m_2_ here,

$$E_{ca}d_{2}=\frac{M_{ca}d_{2}}{M_{ba}}E_{ba}+e_{2}Therefore, e_{2}=E_{ca}d_{2}-\frac{M_{ca}d_{2}}{M_{ba}}E_{ba}=d_{2}(E_{ca}-\frac{M_{ca}}{M_{ba}}E_{ba})$$

Substituting the values for m_2_ and e_2_ in equation 6,

$$E_{ba} =\frac{d_{2}e_{1}+m_{f}d_{2}(E_{\mathrm{ca}}-\frac{M_{\mathrm{ca}}}{M_{\mathrm{ba}}}E_{\mathrm{ba}})}{d_{1}d_{2}-\frac{M_{\mathrm{ca}}d_{2}}{M_{\mathrm{ba}}}m_{f}}=\frac{e_{1}+m_{f}(E_{\mathrm{ca}}-\frac{M_{\mathrm{ca}}}{M_{\mathrm{ba}}}E_{\mathrm{ba}})}{d_{1}-\frac{M_{\mathrm{ca}}}{M_{\mathrm{ba}}}m_{f}}$$

$$Therefore, E_{\mathrm{ba}}d_{1}-\frac{M_{\mathrm{ca}}}{M_{\mathrm{ba}}}m_{f}=e_{1}+m_{f}(E_{\mathrm{ca}}-\frac{M_{\mathrm{ca}}}{M_{\mathrm{ba}}}E_{\mathrm{ba}})$$

$$Therefore, e_{1}={E_{\mathrm{ba}}d_{1}-m}_{f}E_{\mathrm{ca}}$$

Now we have equations for m_1_, m_2_, e_1_ and e_2_ in terms of d_1_, d_2_ and mf.

$m_{1}=d_{1}M_{\mathrm{ba}}-m_{f}M_{\mathrm{ca}}$

$m_{2}=\frac{M_{\mathrm{ca}}d_{2}}{M_{\mathrm{ba}}}$

$e_{1}={E_{\mathrm{ba}}d_{1}-m}_{f}E_{\mathrm{ca}}$

$e_{2}=d_{2}\left( E_{\mathrm{ca}}-\frac{M_{\mathrm{ca}}}{M_{\mathrm{ba}}}E_{\mathrm{ba}} \right)$

**Simulations used for testing the sensitivity and robustness of the predictions:**

For each pathway-prediction combination simulations were run 10,000 times with n = 100 every time randomizing slopes and errors in the following ranges. The input variable had a mean of 100 and standard deviation of 1000. For pathways 1 to 5 all slopes were randomized independent of each other between -2 to +2, error standard deviations were independently randomly selected from a range of 0 to 1000. For pathways 6 and 7, the slopes $m_{1}$ and $m_{2}$ ranged between 0 to 2 and m_f_ ranged between -2 to +2. Other parameter ranges were similar to pathways 1 to 5. To see the sensitivity of null hypothesis rejection, a set of simulations was run at n = 500.

The input variables are generated by random selection from a Gaussian distribution. Other variables are generated using the pathway equations that include errors which are generated randomly from Gaussian distributions with a mean of zero. The parameters of the equations namely slopes, intercepts and error standard deviations are generated by uniform random selection from a wide range. Each simulation is run 10,000 times with a new set of randomly chosen parameters every time. At the end of each run the prediction is tested using an appropriate null hypothesis. The simulation results are represented in a two dimensional parameter space as dots with specific colours describing acceptance of the null or alternative hypotheses as described below. The axes of the parameter spaces can be different for different pathways depending upon their relative importance. The null hypotheses used to test the prediction against simulation results for each of the above four predictions are as follows.
