# Supplementary material for: Inferring causal pathways among three or more variables from steady-state correlations in a homeostatic system
Source: PLoS One. 2018 Oct 11;13(10):e0204755. doi: 10.1371/journal.pone.0204755 (PMC6181337; doi:10.1371/journal.pone.0204755)
Supplement: S2 Text — (DOCX) [file pone.0204755.s002.docx]

**Supporting Information**

**S2 Text. The pre-diabetic state: Estimating causal parameters from published empirical data.**

Estimates for most of the parameters used in the prediabetic steady state model are available in literature and we use them as under.

**Table 1:** Symbols used in the equations and the parameter ranges used in the simulations.

| **Symbol** | **Description** | **Estimated Mean value used for the parameters (units)** |
| --- | --- | --- |
| $d$ | Rate constant for insulin degradation | 0.15  /min |
| $k_{3}$ | Rate constant for glucose-stimulated insulin secretion | 0.08µIU/mg/min |
| $L$ | liver glucose production independent of insulin | 20 mg/dl/min |
| $k_{1}$ | Rate constant for glucose feedback | 0.5 |
| $k_{2}$ | Rate constant for insulin feedback | 2 |

1. $k_{3}$(Rate constant for glucose-stimulated insulin secretion)

The value for this parameter was calculated based on experiments on isolated human pancreatic islets which show glucose-stimulated insulin secretion^1-3^. Insulin secretion was measured in this study after exposing the islet cells to different concentrations of glucose. The mean value of 0.08 was used for this parameter (1). Similar studies have also been performed in rat islets (2) and in vivo as well (3). It is known that in humans, insulin secretion is stimulated by glucose above a threshold estimated to be 63mg/dl. This is why the index HOMA-β has a denominator as glucose concentration above the threshold.

2. $d$(Insulin degradation rate)

The half-life of insulin has been determined experimentally in various model systems ranging from isolated cells to humans. To calculate the rate constant for insulin degradation, we used the half-life of insulin estimated as 5 to 6 minutes (4,5).

3. $L$ (Rate constant for liver glucose production independent of insulin)

The net hepatic glucose production has been measured using a variety of tracer techniques and is reported to be around 10-15mg/dl (6,7). Absence of insulin signalling, such as during extreme hypoglycemia results in a 25-30% increase in hepatic glucose production (8,9). Hence the value of $L$ used in the simulations was 20mg/dl.

1. Since no genuine measure of insulin sensitivity independent of insulin and glucose measurement is available we assume the normal healthy insulin sensitivity to be unity. At the normal level of insulin sensitivity the reduction in liver glucose production mentioned above is brought about by a fasting insulin level of 5 to 10 µIU. The rate constant *K_2_* for insulin feedback can be calculated from this to range between 0.5 and 1. Given these estimates the constant for glucose feedback *K_1_* needed to give the normal fasting value of insulin was calculated as 0.15 to 0.2.
2. As a countercheck to our estimates of K1 and K2, we calculated the normal glucose uptake by tissues based on these parameters, which ranges between 15 to 20 mg/dl/min. Fludeoxyglucose (FDG)-Positron emission tomography (PET) scanning has been used to measure the glucose uptake in specific muscles. Based on these results, the average whole body muscle glucose uptake is close to 18 mg/dl (10–12). These estimates match fairly well.

Thus we use a set of parameters that closely approximate the real life values. However, it needs to be realized that many of the inferences drawn from the prediction signatures are independent of the actual parameters used. Therefore even if some of the parameter estimates used are biased or unrealistic, it is not a serious threat to the conclusions.

**Supporting References**

1. Marchetti P, Scharp DW, Mclear M, Gingerich R, Finke E, Olack B, et al. Pulsatile insulin secretion from isolated human pancreatic islets. Diabetes. 1994;43(6):827–30.

2. Westerlund J, Bergsten P. Glucose Metabolism and Pulsatile Insulin Release From Isolated Islets. Diabetes. 2001 Aug;50(8):1785–90.

3. Chirieac D V, Chirieac LR, Corsetti JP, Cianci J, Sparks CE, Sparks JD. Glucose-Stimulated Insulin Secretion Suppresses Hepatic Triglyceride-Rich Lipoprotein and Apo B Production In Vivo. Diabetes. 2000;49(5):A281.

4. Tomasi T, Sledz D, Wales JK, Recant L. Insulin half-life in normal and diabetic subjects. Rev Neuropsychiatr Infant [Internet]. 1966 Dec;14(12):315–7. Available from: http://www.ncbi.nlm.nih.gov/pubmed/5988016

5. Matthews DR, Hosker JP, Rudenski AS, Naylor BA, Treacher DF, Turner RC. Homeostasis model assessment: insulin resistance and ?-cell function from fasting plasma glucose and insulin concentrations in man. Diabetologia [Internet]. 1985 Jul;28(7):412–9. Available from: http://link.springer.com/10.1007/BF00280883

6. Rothman DL, Magnusson I, Katz LD, Shulman RG, Shulman GI. Quantitation of hepatic glycogenolysis and gluconeogenesis in fasting humans with 13C NMR. Science. 1991 Oct;254(5031):573–6.

7. Shulman GI, Rothman DL, Jue T, Stein P, DeFronzo RA, Shulman RG. Quantitation of muscle glycogen synthesis in normal subjects and subjects with non-insulin-dependent diabetes by 13C nuclear magnetic resonance spectroscopy. N Engl J Med. 1990 Jan;322(4):223–8.

8. Moore MC, Connolly CC, Cherrington a D. Autoregulation of hepatic glucose production. Eur J Endocrinol. 1998;138(3):240–8.

9. Chu CA, Sindelar DK, Neal DW, Donahue EP, Cherrington AD. Comparison of the direct and indirect effects of epinephrine on hepatic glucose production. J Clin Invest. 1997;99:1044–56.

10. Nuutila P, Peltoniemi P, Oikonen V, Larmola K, Kemppainen J, Takala T, et al. Enhanced stimulation of glucose uptake by insulin increases exercise-stimulated glucose uptake in skeletal muscle in humans: studies using [15O]O2, [15O]H2O, [18F]fluoro-deoxy-glucose, and positron emission tomography. Diabetes. 2000 Jul;49(7):1084–91.

11. Hällsten K, Virtanen KA, Lönnqvist F, Sipilä H, Oksanen A, Viljanen T, et al. Rosiglitazone but not metformin enhances insulin- and exercise-stimulated skeletal muscle glucose uptake in patients with newly diagnosed type 2 diabetes. Diabetes. 2002;51(12):3479–85.

12. Fujimoto T, Kemppainen J, Kalliokoski KK, Nuutila P, Ito M, Knuuti J. Skeletal muscle glucose uptake response to exercise in trained and untrained men. Med Sci Sports Exerc. 2003;35(5):777–83.
